# Fermented fruit state shapes flavor quality and methanol accumulation in kiwi wine: A comparative study of three flesh-colored kiwifruit cultivars

**DOI:** 10.1016/j.fochx.2026.104583

**Published:** 2026-10-05

**Authors:** Jiahui Shao, Shiying Huang, Yuzhe Lou, Shiguo Chen, Xingqian Ye, Huan Cheng

**Affiliations:** a College of Biosystems Engineering and Food Science, National-Local Joint Engineering Research Center of Intelligent Food Technology and Equipment, Zhejiang Key Laboratory of Agri-Food Resources and High-Value Utilization, Zhejiang University, Hangzhou 310058, China; b Zhejiang Xiyingtian Technology Co., Ltd, China; c Zhejiang University Zhongyuan Institute, Zhengzhou, 450000, China

**Keywords:** Kiwi wine, Cultivar, Fermentation, Volatile compounds, Methanol, Flavor

## Abstract

The fermented fruit state represents a critical processing variable that simultaneously governs flavor development and methanol accumulation in fruit wine. In this study, three kiwifruit cultivars differing in flesh colors were fermented under four fruit states (peel presence/absence and pulp maceration/juice fermentation), following a 3 × 4 factorial design. Relative to juice fermentation, pulp maceration reduced total acidity and elevated total phenolic and flavonoid contents. A total of 130 volatile compounds were identified across all treatments, with alcohols and esters as the dominant classes. Pulp maceration generally favored ester accumulation and fruity and floral aroma attributes, whereas peeled or juice-fermented wines were characterized by higher contributions of volatile alcohols. Methanol accumulation increased with pulp maceration, and was accompanied by greater water-soluble pectin consumption, higher total pectin degradation, and elevated pectin methylesterase activity. Cultivar background further modulated these responses. These findings provide a practical basis for kiwi wine fermentation strategies.

## Highlights

• A total of 130 volatile compounds were identified in kiwi wines.
• Pulp maceration generally increased the accumulation of esters.
• Pulp contact generally increased phenolic extraction and bitterness perception.
• Methanol accumulation increased with net pectin loss and endpoint PME activity.

## 1 Introduction

Kiwifruit (*Actinidia* spp.) is an economically important fruit crop worldwide, valued for its juicy texture, distinctive flavor, and exceptional nutritional quality (Zhang et al., 2023; Zheng, Luo, et al., 2025). Global kiwifruit production has increased steadily in recent years, surpassing 4.43 million tons annually, with China accounting more than 50% of the total supply (FAO, 2023). Despite this abundance, kiwifruit is a highly climacteric fruit prone to postharvest softening, which results in considerable post-harvest losses and places significant pressure on supply chains. Processing therefore represents an essential strategy for reducing fruit waste and improving resource utilization. Compared with conventional products such as juice, jam, and dried fruit, kiwi wine offers additional advantages: alcoholic fermentation extends shelf life, generates diverse flavor metabolites, and substantially elevates both flavor complexity and commercial value.

Flavor quality is widely regarded as a primary determinant of consumer acceptance in fruit wines. Previous studies have explored the influence of yeast strains, inoculation strategies, clarification treatments, and cultivar selection on the flavor and nutritional properties of kiwi wine (Huang et al., 2024; Sun et al., 2024, Sun et al., 2025; Wei et al., 2025). Alongside flavor, methanol content represents a critical quality and safety parameter. Excessive methanol can compromise flavor purity and sensory acceptability even at sub-toxic concentrations, and at elevated levels poses serious health risks, including blindness and fatality (Shen et al., 2024). Despite its importance, methanol formation during kiwi wine fermentation has received comparatively little systematic attention. Methanol accumulation in fruit wines is closely associated with pectin content, degree of esterification, and pectinolytic enzyme activities, particularly pectin methylesterase (PME), which catalyzes the hydrolysis of pectin methoxyl groups to release methanol (Han & Du, 2022). Kiwifruit is rich in pectin, particularly in peel and pulp tissues (Qi et al., 2019), and the release and enzymatic degradation of pectic materials during fermentation can therefore provide substrates for methanol formation (Miljić et al., 2016). However, existing research on methanol in fruit wines primarily focuses on the effects of pectinase addition, fruit maturity, and physicochemical processing parameters, while the relationships among net changes in pectin fractions, endpoint pectinolytic enzyme activities, and methanol concentrations remain insufficiently characterized (Han & Du, 2022; Miljić et al., 2016; Shen et al., 2024).

A key yet underexplored variable in kiwi wine production is the fermented fruit state, which is defined here by two processing factors: (i) peel presence/absence (peeled or unpeeled before juicing) and (ii) fermentation matrix (pulp maceration or juice fermentation). Peel and pulp are not only major reservoirs of pectin but also important sources of volatile aroma compounds, phenolics, and flavor precursors (Gao et al., 2024; Liang et al., 2021). Consequently, the fermented fruit state may simultaneously shape flavor compound release and methanol formation, creating an inherent trade-off between aroma enhancement and safety control. This complexity is further compounded by cultivar diversity: kiwifruit cultivars differing in flesh color commonly exhibit distinct aroma precursor profiles, phenolic compositions, and cell wall structural characteristics, which may lead to divergent responses to the same fermentation condition (Wei et al., 2024; Zhao et al., 2025; Zhou et al., 2025). Nevertheless, it remains unclear how the fermented fruit state simultaneously regulates flavor development and methanol accumulation, and whether these effects are consistent across kiwifruit cultivars with different flesh color backgrounds.

To address these gaps, this study evaluated three commercial kiwifruit cultivars with contrasting flesh colors— Hongyang (red), Jinyan (yellow), and Xuxiang (green) — fermented under four fruit states: peeled juice fermentation, peel-involved juice fermentation, peeled pulp maceration fermentation, and peel-involved pulp maceration fermentation. By integrating volatile compound profiling, electronic nose and tongue analysis, sensory evaluation, pectin fraction quantification, pectinolytic enzyme activity measurements, and methanol accumulation, this study aimed to elucidate how peel and pulp involvement regulate flavor development, pectin degradation, and methanol formation. These findings provide insight into the balance between flavor enhancement and methanol control, offering a theoretical basis for optimizing kiwi wine fermentation and improving product quality.

## 2 Materials and methods

### 2.1 Experimental materials

Three commercial kiwifruit cultivars were used in this study: Hongyang (HY, red-fleshed), Jinyan (JY, yellow-fleshed), and Xuxiang (XX, green-fleshed). All fruits were harvested from Cangxi County, Guangyuan City, Sichuan Province. Detailed physicochemical characteristics of the kiwifruit samples are provided in Table S1. *Saccharomyces cerevisiae* AQ, used as the fermentation starter, was obtained from Angel Yeast Co., Ltd. (Hubei, China).

Rutin, ethanol, methanol, and other analytical grade reagents were purchased from Shanghai Yuanye Biochemical Technology Co., Ltd. (Shanghai, China). The mixture of n-alkanes (C_10_-C_25_) was purchased from Shanghai Aladdin Biotechnology Co., Ltd. (Shanghai, China). Kits for the determination of pectin fractions and enzyme activities were ordered from Suzhou Grace Biotechnology Co., Ltd. (Suzhou, China).

### 2.2 Kiwi wine preparation

To ensure sample uniformity across treatments, kiwifruits with comparable firmness values of 3–5 N were selected through a texture analyzer (Stable Micro Systems, UK). Selected fruits were thoroughly washed and divided into two groups: peeled and unpeeled. Each group was then pressed using a laboratory juicer (Midea Co., Ltd., China), and the resulting extract was either filtered to obtain juice or left unfiltered to obtain mash containing pulp solids. Combining the peeling and filtration variables yielded four fermented fruit states: peeled juice fermentation (QPJ), peel-involved juice fermentation (DPJ), peeled pulp maceration fermentation (QPL), and peel-involved pulp maceration fermentation (DPL). The full preparation workflow is illustrated in Fig. 1.

**Fig. 1:**
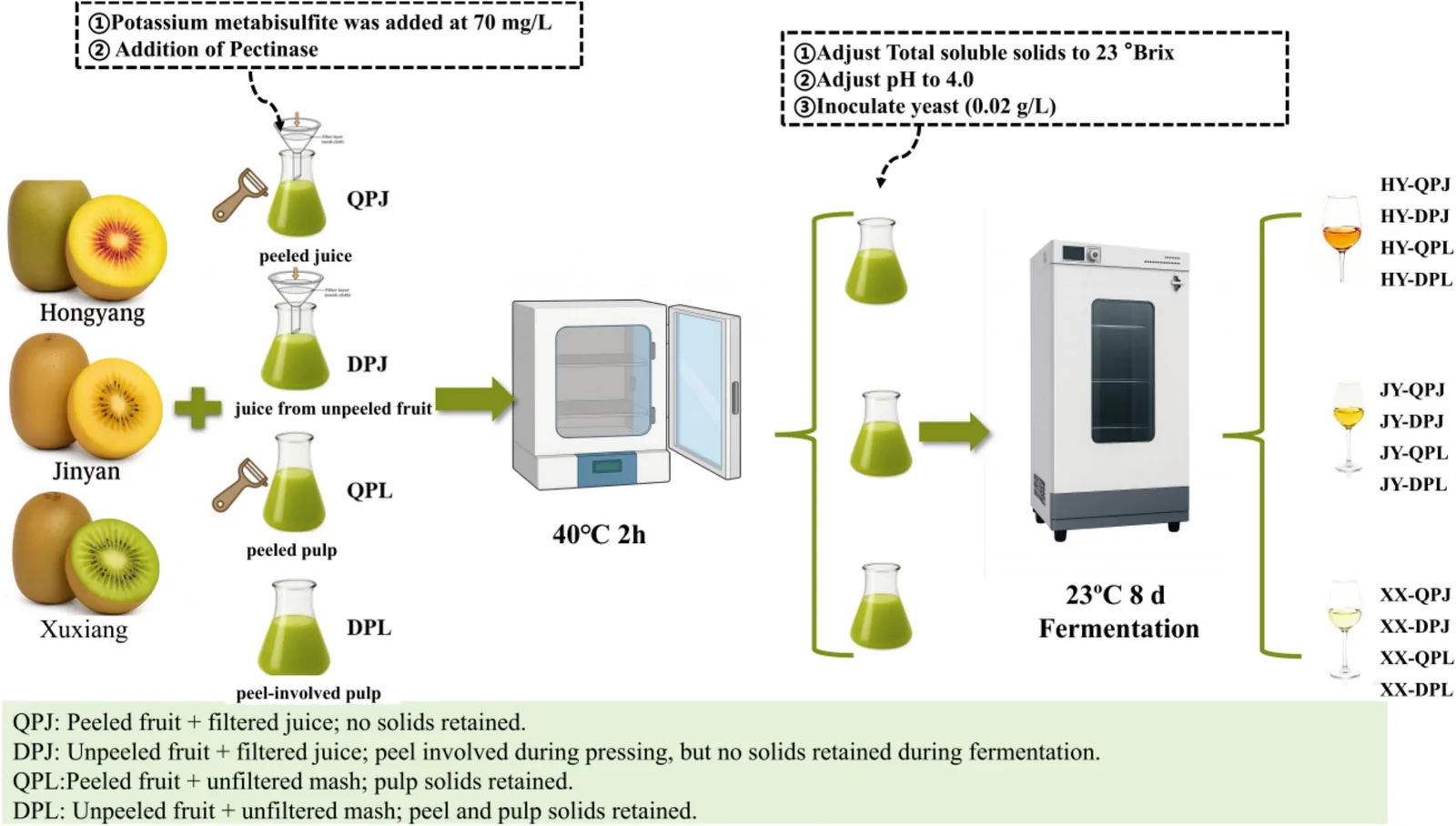
Schematic illustration of the fermentation process of kiwi wine. HY, Hongyang; JY, Jinyan; XX, Xuxiang; QPJ, peeled juice fermentation; DPJ, peel-involved juice fermentation; QPL, peeled pulp maceration fermentation; DPL, peel-involved pulp maceration fermentation.

Fermentation conditions were established based on preliminary trials and standard industrial practices. Potassium metabisulfite was added at 70 mg/L, followed by pectinase treatment (100 mg/L; Xiasheng, China) at 40 °C for 2 h. Notably, all treatments received the same dose of commercial pectinase, a common pretreatment for fruit wine production. All subsequent results are interpreted in the context of commercial pectinase pretreatment, as no pectinase-free control was included. The soluble solids content was then adjusted to 23°Brix by adding sucrose, and the pH was adjusted to 4.0. Active dry yeast was then inoculated at 0.02 g/L to initiate fermentation, which was conducted at 23 ± 1 °C for 8 d. After fermentation, samples were filtered through sterile gauze, centrifuged at 8000 rpm for 5 min, and stored at 4 °C until analysis. The experiment followed a 3 × 4 factorial design encompassing three kiwifruit cultivars and four fermented fruit states, with three independent fermentation replicates prepared per treatment.

### 2.3 Physicochemical and functional properties

Basic physicochemical parameters of kiwi wine, including reducing sugar, total acid, pH, total soluble solids (TSS), and alcohol content, were measured according to previous studies with slight modifications (Achhami et al., 2025; Bayoï & Etoa, 2024; Lan et al., 2024). Reducing sugar content was measured using the DNS method. Total acid was determined by titration with 0.1 mol/L NaOH. TSS was measured using a Brix meter (LH-B55, Lohand Biological Co., Ltd., Hangzhou, China). The pH value was determined using a calibrated pH meter (PHS-25-3C, Leici Co., Ltd., Shanghai, China). Alcohol content was determined using distillation followed by alcoholmeter measurement (CJ-JJ1, Chuangjimei Co., Ltd., Hebei, China) and expressed as % (*v*/*v*).

Total phenolic content (TPC) was quantified using the Folin-Ciocalteu method, following Liang et al. (2024) with minor modifications. Briefly, 0.5 mL of the sample (diluted 1:10, *v*/*v*) was mixed with Folin-Ciocalteu reagent and water, after which 7.5% (*w*/*v*) Na_2_CO_3_ solution was added. The mixture was incubated under light-protected conditions for 2 h, and absorbance was measured at 760 nm. TPC was calculated from a gallic acid calibration curve and expressed as mg gallic acid equivalents per liter (mg GAE/L).

Total flavonoid content (TFC) was quantified according to Liu et al. (2022) with minor modifications. A 0.5 mL aliquot of the sample (diluted 1:10, *v*/*v*) was combined with 0.25 mL of 5% *w*/*v* NaNO_2_ and allowed to react for 5 min. Subsequently, 5 mL of 5% (*w*/*v*) AlCl₃ solution was added and the reaction was continued in the dark for an additional 5 min. Finally, 2 mL of 2 mol/L NaOH was introduced, and the mixture was incubated in the dark for 10 min before absorbance measurement. TFC was calculated from a rutin standard curve and reported as mg rutin equivalents per liter (mg RE/L).

### 2.4 HS-SPME-GC–MS and OAV analysis

#### 2.4.1 HS-SPME-GC–MS analysis

Volatile compounds in kiwi wine were analyzed using headspace solid-phase microextraction (HS-SPME) coupled with gas chromatography–mass spectrometry (GC–MS), following the method described by Hao et al. (2025). Briefly, 5 mL of the wine sample was placed in a 20 mL vial, followed by the addition of 10 μL 2-octanol and 1 g NaCl, and then sealed with a silicone septum. After equilibration at 45 °C for 15 min, the SPME fiber was exposed to the vial headspace at 45 °C for 25 min with continuous stirring at 200 rpm. After extraction, the fiber was immediately inserted into the GC injection port for thermal desorption for 5 min. Volatile compounds were separated on a WAX capillary column (30 m × 0.25 mm × 0.25 μm; Agilent, CA, USA). The oven temperature was initially held at 40 °C for 2 min, increased to 80 °C at 3 °C/min, to 105 °C at 2 °C/min, to 180 °C at 3 °C/min, and finally to 250 °C at 10 °C/min, holding for 1 min. The transfer line, ion source, and quadrupole temperatures were maintained at 280 °C, 230 °C, and 150 °C, respectively. Volatile compounds were identified by comparing mass spectra with the NIST11 library and by calculating retention indices using n-alkanes (C_10_-C_25_). The content of volatile compounds was calculated as follows:

$$\text{Volatile compound content}\left(\mathrm{\mu g}/L\right)=\frac{S_{0}\times C_{1}}{S_{1}}$$

where:

(1) $$S_{0}\;\text{is the peak area of volatile substances}$$

(2) $$S_{1}\;\text{is the peak area of}\;2-\text{octanol}$$

(3) $$C_{1}\;\text{is the content of}\;2-\text{octanol}$$

Volatile concentrations were estimated semi-quantitatively using 2-octanol as a single internal standard, and should therefore be interpreted as approximate values.

#### 2.4.2 Odor activity value (OAV) analysis

To evaluate the contribution of each volatile flavor compound in kiwi wine, OAV analysis was performed (Ayestarán et al., 2019).

$$\mathrm{OAV}=\frac{C_{i}}{OT_{i}}$$

where C_i_ is the concentration of the individual compound; OT_i_ is the perception threshold of the individual compound. The odor thresholds were taken from the literature; therefore, the calculated OAVs should be regarded as approximate.

### 2.5 Gas chromatography electronic nose (*E*-nose) analysis

Heracles II E-nose (Alpha M.O.S., Toulouse, France) was used to analyze the aroma profiles of samples. The instrument consisted of an autosampler, MXT-5 and MXT-1701 capillary columns, and two flame ionization detectors (FIDs). Based on reference and manufacturer recommendations (Jo et al., 2025), 2 mL sample was added into a 20 mL vial for analysis and equilibrated at 40 °C. Subsequently, 2500 μL of headspace gas was injected into the GC inlet at 250 °C. A 20 μL aliquot of the n-alkane mixture (C_6_-C_16_) was transferred into a headspace vial and analyzed under the same conditions as the samples to calculate retention indices (RIs). Chromatographic peaks showing differences among samples were identified by comparing their RIs with reference values in the AroChemBase database.

### 2.6 Electronic tongue (*E*-tongue) analysis

Following the method described by Zheng, Sheng, et al. (2025), kiwi wines were collected for taste analysis using an E-tongue (Astree II V5, Alpha MOS, France). For each measurement cycle, 80 mL kiwi wine sample and a cleaning solution were placed in 120 mL beakers and alternately loaded into the autosampler. Sensor response data were acquired at 1 Hz for 120 s per solution, followed by a 10 s cleaning cycle. Each sample was analyzed in seven technical replicates, and the mean sensor response values obtained during the last 20 s of the final three replicates were used for statistical analysis.

### 2.7 Sensory evaluation

Quantitative descriptive analysis (QDA) was conducted following previously published methods (Araujo et al., 2021; Jiménez-Pulido et al., 2024; Kong et al., 2019). A sensory panel of 15 members (8 females and 7 males, aged 23–28 years) with previous experience in sensory evaluation was recruited. The panelist screening criteria were based on ISO 8586 and their discrimination ability and repeatability were assessed after training. Before formal evaluation, panelists attended training sessions twice a week for one month, focusing on the identification and description of the sensory attributes of kiwi wine. During preliminary sample evaluations, panelists proposed descriptors and reached a consensus on the final descriptors and their definitions. Approximately 30 mL of each sample was served in transparent tasting glasses, labeled with random codes, and presented in a randomized order. Panelists rated each attribute on a 9-point intensity scale (1 = low, 9 = high).

### 2.8 Methanol content and pectin fraction determination

Methanol content was determined by gas chromatography with flame ionization detection (GC-FID; Agilent G2790A, CA, USA), following the procedure described by Han and Du (2022) with appropriate modifications. Prior to analysis, samples were subjected to distillation. Methanol was quantified using 2-methyl-2-butanol as an internal standard via the internal standard calibration method and expressed as mg/L. Method validation assessed linearity, LOD, LOQ, precision, and matrix recovery, with procedures and results detailed in Table S2.

Water-soluble pectin (WSP), protopectin, and total pectin contents were determined using commercial assay kits from Suzhou Grace Biotechnology Co., Ltd. (Suzhou, China). Each pectin fraction was extracted from the corresponding sample using the extraction reagents provided in the kits, and results were expressed as mg/mL.

To assess pectin degradation occurring during fermentation, pectin fractions were quantified at two time points: day_0_ (after pectinase pretreatment and prior to yeast inoculation) and day_8_ (at the end of fermentation). Changes in pectin fractions were calculated as:

ΔPectin = Pectin_day 0_-Pectin_day 8_.

### 2.9 Determination of apparent pectinolytic activities

The apparent activities of pectin methylesterase (PME), polygalacturonase (PG), and pectin lyase (PL) were determined using commercial assay kits (Suzhou Grace Biotechnology Co., Ltd., Suzhou, China). Enzyme activities were calculated based on the standard curves supplied with the kits and expressed according to the protocols. All measurements were performed on fermentation samples collected at day_8_. Since all treatments received identical pectinase pretreatment prior to fermentation, the activities measured at this endpoint reflect the residual and microbially modulated enzymatic activity within the fermented wine matrix; these are therefore defined as apparent pectinolytic activities.

### 2.10 Statistical analysis

Data are presented as mean ± standard deviation of three independent biological replicates. ANOVA was performed to evaluate the individual and interactive effects of kiwifruit cultivar and fermented fruit state on measured parameters, with significance set at *p* < 0.05 (Table S3). OPLS-DA and partial least squares (PLS) regression were performed using SIMCA 14.1. Pearson correlations were calculated using biological replicate data (*p* < 0.05). HCA used *Z*-score-standardized data, Euclidean distance, and complete linkage. Entropy-weighted TOPSIS integrated total OAV, sensory fruity and floral intensities, bitterness, and methanol concentration to rank the 12 treatments (Table S4). Other statistical analyses were performed using IBM SPSS Statistics 27.0.1.0, and figures were generated using Origin 2024.

## 3 Results and discussion

### 3.1 Physicochemical parameters and functional components

#### 3.1.1 Physicochemical properties analysis

The physicochemical properties of kiwi wine produced under the four fermented fruit states are summarized in Fig. 2. Alcohol contents ranged from 7.5% to 8.5% (*v*/*v*) across all treatments (Fig. 2A), confirming that comparable degrees of alcoholic fermentation were achieved. This consistency provides a reliable basis for attributing observed differences in acid and sugar profiles primarily to fermented fruit state rather than fermentation efficiency. Pulp maceration fermentation generally yielded wines with higher residual reducing sugar and lower total acid compared with juice fermentation. This trend was most pronounced in DPL wines, which showed the lowest total acid (9.86–13.29 g/L, Fig. 2B) and the highest reducing sugar content (4.59–8.39 g/L, Fig. 2C). In contrast, DPJ wines showed relatively higher acidity and lower reducing sugar. These results suggest that solid-tissue contact, particularly pulp maceration, substantially influences acid and sugar retention during fermentation. One likely explanation is that pulp-derived polysaccharides and structural components are not readily metabolized by yeast, while the elevated nitrogen content of pulp may alter yeast metabolic activity, ultimately modifying sugar utilization and organic acid production (Tu et al., 2025; Zhao et al., 2025; Zheng, Luo, et al., 2025). Peel contact further modified the sugar profile within the pulp maceration group. For example, HY-DPL contained 8.39 g/L reducing sugar, compared with 5.20 g/L in the corresponding peeled treatment HY-QPL. This increase may reflect the release of compounds from peel, including phenolics, polysaccharides, and minerals, which could increase matrix complexity and affect sugar utilization (Mekoue Nguela et al., 2019).

**Fig. 2:**
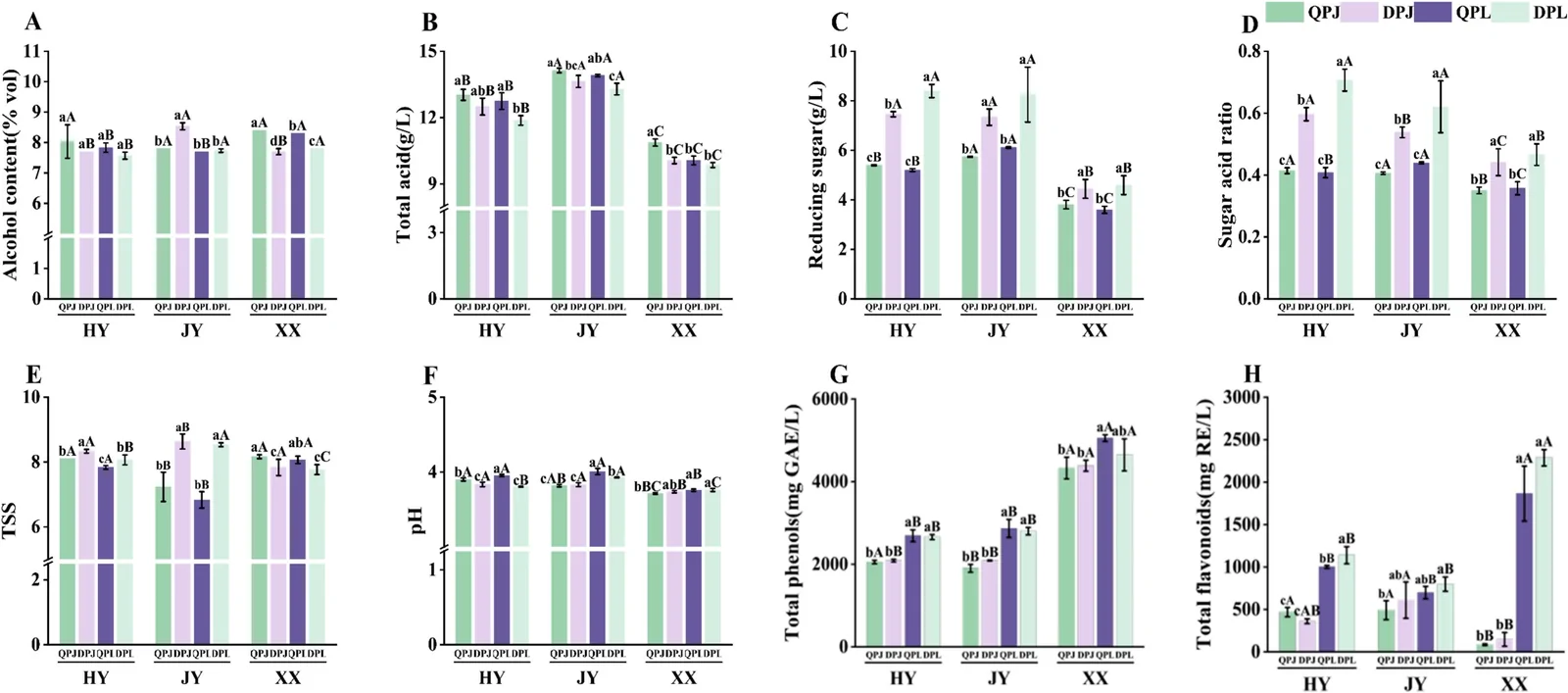
Physicochemical and functional properties of kiwi wines produced under different fermented fruit states: (A) alcohol content (% *v*/*v*); (B) total acid (g/L); (C) reducing sugar (g/L); (D) sugar-acid ratio; (E) total soluble solids (TSS, °Brix); (F) pH; (G) total phenol content (mg GAE/L); (H) total flavonoid content (mg RE/L). Different lowercase letters indicate significant differences (*p* < 0.05) among fruit states within the same cultivar; different uppercase letters indicate significant differences (*p* < 0.05) among cultivars under the same fruit state. Letters should be interpreted only within their respective comparison sets.

Cultivar-dependent differences were also observed. JY wines tended to have higher acidity and reducing sugar, HY wines showed a relatively higher sugar-acid ratio, and XX wines generally exhibited lower levels of both acid and sugar. These observations are consistent with Chen et al. (2024), who demonstrated that cultivar-specific sugar-acid composition creates distinct fermentation matrices for kiwifruit fermentation. Taken together, fermented fruit state affected the sugar-acid characteristics of kiwi wine. These responses also varied among cultivars.

#### 3.1.2 Functional components analysis

Polyphenols and flavonoids are key contributors to the functional properties and sensory quality of kiwi wines (Minussi et al., 2003). Compared with juice fermentation, pulp fermentation resulted in markedly higher TPC, ranging from 2657.27 to 5052.67 mg GAE/L, whereas juice-fermented wines ranged from 2053.23 to 4384.33 mg GAE/L (Fig. 2G). A parallel trend was observed for TFC (Fig. 2H), demonstrating that fruit solids served as an important source of extractable phenolics and flavonoids. Pulp maceration likely facilitates their release by extending solid-liquid contact during fermentation and promoting the extraction of soluble and phenolic compounds from fruit pulp tissues (Cheng et al., 2024; Pascual et al., 2016).

The influence of peel contact on TPC and TFC followed distinct patterns depending on the fermentation system. In juice fermentation, TPC was slightly elevated in peel-involved treatments relative to peeled ones, which aligns with the well-established enrichment of polyphenols in fruit peel (Liu et al., 2018). In pulp maceration fermentation, however, peeled samples exhibited slightly higher TPC than their peel-involved counterparts (e.g., JY-QPL: 2865.35 mg GAE/L, JY-DPL: 2800.71 mg GAE/L). This result may be attributed to oxidative reactions during maceration, where phenolics released from solid tissues are oxidized by polyphenol oxidase or peroxidase and subsequently polymerized or bound to proteins and polysaccharides, thereby reducing the soluble and detectable TPC (Illera et al., 2019). In contrast, TFC was enhanced by peel involvement in both juice and pulp fermentation systems, in line with the findings of Cosmulescu et al. (2015), who reported that peel is an important source of flavonoids.

Among the three flesh-colored cultivars, XX wine consistently exhibited the highest TPC and TFC under pulp maceration, indicating a richer reservoir of phenolic and flavonoid compounds. These differences may partly reflect cultivar-related variation in phenolic composition or metabolism, but this explanation remains hypothetical and was not directly tested in this study (Li et al., 2018; Zhao et al., 2022; Zhou et al., 2023). Overall, these findings indicate that pulp fermentation was associated with higher TPC and TFC, while the effect of peel treatment on TPC and TFC depended on the fermentation matrix.

### 3.2 Volatile profiles and OAV analysis

#### 3.2.1 Volatile profile of kiwi wine identified by HS-SPME-GC–MS

As shown in Table S5, a total of 130 volatile compounds were identified in all kiwi wine samples, including 39 alcohols, 42 esters, 13 acids, 5 aldehydes, 6 ketones, 6 terpenes, and 19 other compounds. Alcohols and esters were the dominant volatile categories, suggesting that they formed the main chemical basis for the alcoholic, fruity, and floral aroma characteristics of kiwi wine. Among them, 42 compounds were shared by the three cultivars, and 47 compounds were shared across the fruit states. These common compounds represented the basic volatile framework of kiwi wine.

Alcohols, especially higher alcohols, are mainly produced through amino acid catabolism via the Ehrlich pathway and play a dual role in wine flavor (Qu et al., 2024). Alcohols represented the largest proportion of total volatiles in most kiwi wine samples, accounting for 39.48–81.55% (Fig. 3). The corresponding semi-quantitative concentrations of the major volatile compound classes (μg/L) are provided in Table S6. The alcohol profile was dominated by 3-methyl-1-butanol, followed by 2-methyl-1-propanol, 1-propanol, and phenethyl alcohol. These compounds are commonly associated with fermented, alcoholic, malty, and floral aroma notes. The accumulation of volatile alcohols varied with cultivar background and fruit state. Peeled juice fermentation resulted in higher relative proportions of volatile alcohol levels than the others, possibly because phenolics, polysaccharides, and mineral elements from peel altered amino acid availability and yeast nitrogen metabolism, thereby limiting the conversion of amino acids into α-keto acid precursors for higher alcohol formation (Rollero et al., 2021). In contrast, XX wines showed higher relative proportions and total contents of alcohols (Fig. 3C), suggesting a stronger alcoholic aroma background.

**Fig. 3:**
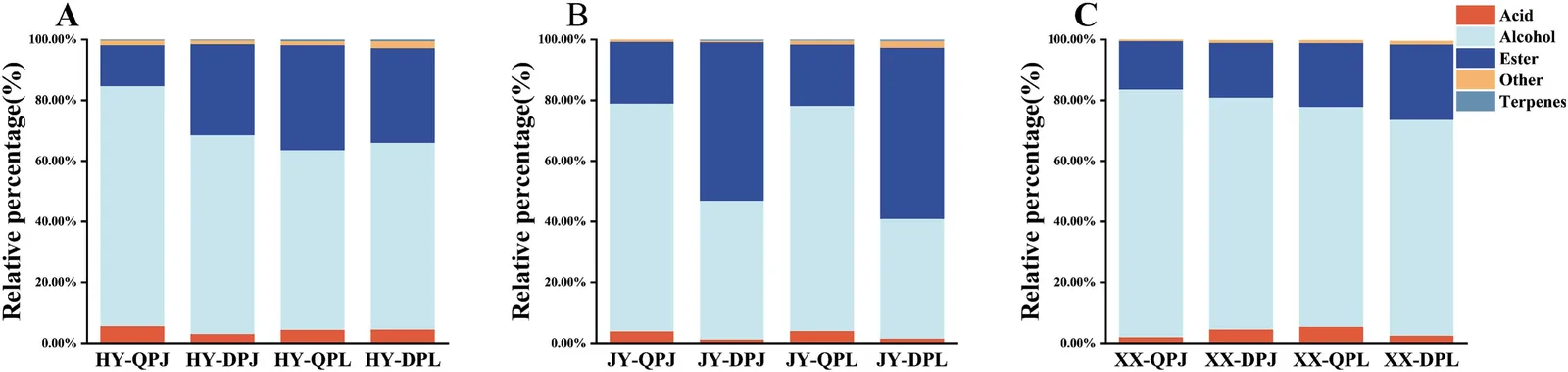
Relative proportions of volatile compound classes in kiwi wines produced under different fermented fruit states: (A) HY wine; (B) JY wine; (C) XX wine.

Esters are important aroma compounds in fruit wines, mainly formed through yeast-mediated reactions between alcohols and fatty acids, and contribute fruity, floral, and sweet notes (Qu et al., 2024). In this study, isobutyl acetate, isoamyl acetate and phenethyl acetate were detected in all 12 kiwi wines, forming a stable ester framework. Pulp-fermented wines generally showed higher total ester proportions (20.13–56.37%) than juice-fermented wines (13.46–52.23%), and ethyl caprylate was more abundant in pulp-fermented samples, potentially enhancing fruity and sweet aroma fullness. This may be attributed to pulp maceration increasing the availability of ester precursors, such as fatty acids, amino acids, and alcohols, while modulating the metabolism of yeast (Rollero et al., 2021). Ethyl benzoate and benzyl acetate were mainly detected in XX wines, possibly related to cultivar-specific differences in aromatic amino acid metabolism, pathways, and precursors (Prusova et al., 2022). Overall, pulp maceration tended to enhance ester aroma potential.

Volatile acids in fruit wines originate from both raw materials and yeast metabolism and can serve as important precursors for ester formation (Sousa et al., 2020). Acetic acid and octanoic acid were the major volatile acids in kiwi wines. Pulp-fermented wines generally showed higher acetic acid levels, probably because pulp maceration increased contact of solid pulp, promoted the release of acid precursors, and modified oxygen transfer and yeast metabolism (Furdíková et al., 2019). Peeled treatments also tended to show higher volatile acid contents, which may favor sugar and fatty acid metabolism and increase volatile acid production (Zhou et al., 2023). Among cultivars, HY wines showed higher volatile acid contents (310.28–1392.63 μg/L) and proportions (4.02–5.63%) than JY and XX wines, suggesting a stronger acid-related aroma background.

#### 3.2.2 Analysis of OAV for potential key aroma compounds in kiwi wine

Even at low concentrations, certain volatile compounds may significantly influence the overall aromatic profile of samples (Wang et al., 2025). Based on OAV analysis (Table S7), 12 compounds with OAV > 1.0 were identified in at least one treatment. Esters and alcohols were the main active groups, indicating that fruity and alcoholic notes were the major aroma-active dimensions of kiwi wine. Among these compounds, Isoamyl acetate showed the highest OAV among all key active compounds. This indicates that isoamyl acetate was the dominant contributor to the fruity aroma of kiwi wine. In contrast, 3-methyl-1-butanol also exhibited relatively high OAV, ranging from 11.45 to 45.36. This compound mainly contributed to the alcoholic and cheese-like aroma. This finding agrees with previous studies that identified isoamyl acetate and 3-methyl-1-butanol as key aroma-active compounds contributing to the characteristic aroma of kiwi wine (Yang et al., 2025).

Higher OAVs of 3-methyl-1-butanol were observed in several peeled or juice fermentation samples, indicating a stronger contribution of alcoholic and fermented aroma notes. In contrast, some ester compounds showed higher aroma contributions in peel-involved or pulp samples. For example, methyl decanoate showed OAV ranging from 1.15 to 3.33, and its contribution was generally higher in several peel-involved or pulp treatments. This may be associated with enhanced fatty acid metabolism and esterification reactions, where greater release of precursors could favor the formation of methyl decanoate and increase its fruity contribution (Xu et al., 2025). These results suggest that fruit state did not simply increase all aroma-active compounds. Instead, it reshaped the relative contribution of fruity and alcoholic aromas.

To further visualize the distribution of key aroma-active compounds, compounds with OAV > 1 were standardized and subjected to hierarchical clustering (Fig. 4). In HY wine, DPL showed higher standardized OAVs for esters such as phenethyl acetate, isoamyl acetate, and methyl decanoate, suggesting that peel and pulp participation may enhance precursor release and esterification reactions. In JY wine, higher standardized OAVs of 1-heptanol and linalool were observed in the peeled groups, indicating that peeling may contribute more to fresh, herbal, and floral aroma characteristics. For XX wine, the standardized OAV profile was characterized by relatively higher alcohols, implying stronger herbal, alcoholic, and fermented notes.

**Fig. 4:**
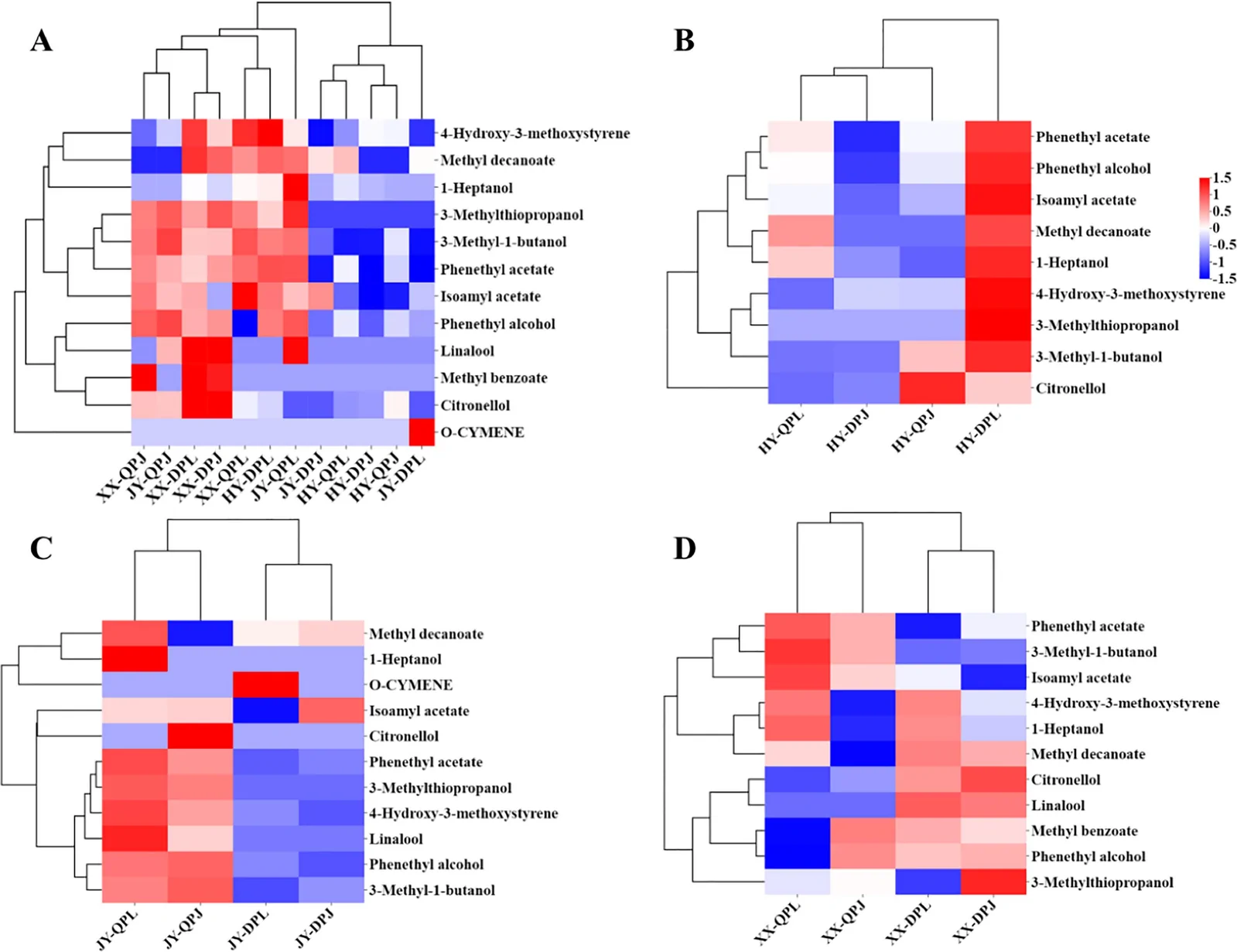
Hierarchical clustering heatmap of key aroma compounds (OAV > 1) in kiwi wine based on Euclidean distance and complete linkage: (A) all treatments; (B) HY wine; (C) JY wine; (D) XX wine.

The clustering also showed a clear separation between peel-involved and peeled treatments, indicating that peel contact markedly affected the aroma profile of wine. Higher standardized OAVs of 3-methyl-1-butanol and phenethyl alcohol were mainly observed in peeled treatments. By contrast, phenethyl acetate, isoamyl acetate, linalool, and citronellol were more prominent in pulp fermentation treatments, indicating that pulp involvement may enhance floral and fruity notes by promoting precursor release and esterification. Collectively, these patterns indicate that fruit state reshaped the aroma profile of kiwi wine.

### 3.3 *E*-nose analysis

An E-nose was employed to provide complementary aroma fingerprint profiling in conjunction with GC–MS analysis. As shown in Fig. 5A, principal component analysis (PCA) revealed a clear separation among samples, with PC1 and PC2 explaining 74.7% and 14.9% of the total variance, respectively. Cultivar background contributed to the overall sample distribution, reflecting inherent differences in volatile precursor pools and cultivar-specific aroma matrices (Chen et al., 2024). However, DPL samples tended to deviate from their cultivar clusters. To provide chemical context, E-nose peaks showing pronounced differences in peak area among treatments were selected for identification by RI matching. Peaks assigned to propyl acetate and methyl pentanoate showed higher responses in HY-DPL and JY-DPL than in the corresponding DPJ treatments, whereas those assigned to 1-propanol and 2-methyl-1-propanol varied among cultivars and fruit states (Table S8). This shift was broadly consistent with the GC–MS and OAV results, in which pulp maceration tended to enhance ester aroma potential, while cultivar background modulated alcohol aroma profiles.

**Fig. 5:**
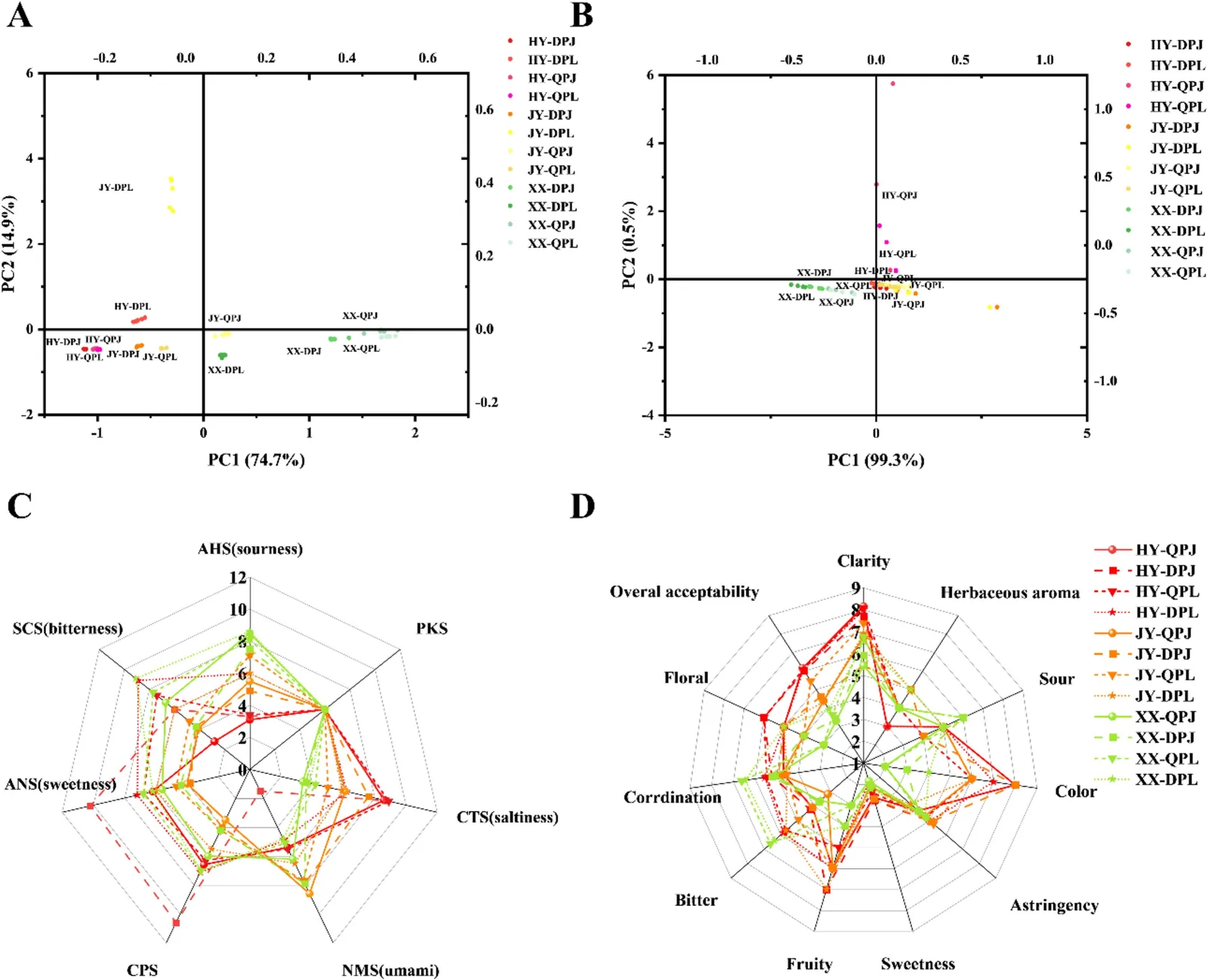
Integrated sensory and electronic sensor analysis: (A) PCA score plot based on E-nose data; (B) PCA score plot based on E-tongue data; (C) radar chart of E-tongue data; (D) radar chart of sensory evaluation results.

To further verify the discrimination among samples, OPLS-DA was constructed (Fig. S1). The model showed good explanatory and predictive performance, with R^2^X, R^2^Y and Q^2^ values of 0.904, 0.835, and 0.748. The permutation test further supported the reliability of the model, with the intersection of the Q^2^ regression line with the vertical axis intercept below 0, suggesting that the model was not overfitted (Fig. S2). DPL samples from all cultivars tended to separate from the other treatments, consistent with the PCA results.

### 3.4 *E*-tongue analysis and sensory evaluation

E-tongue analysis was performed to evaluate the overall taste profiles of kiwi wine. As shown in Fig. 5B, the first two principal components, PC1 (99.3%) and PC2 (0.5%), could reveal most information about the original data. HY and JY wines were closely clustered, whereas XX samples were clearly separated from the others. This indicated that HY and JY wines shared more similar taste profiles. This separation may be partly associated with the higher TPC and TFC of XX wines, which strongly influences sensor responses related to bitterness and astringency (Jagatić Korenika et al., 2023).

The radar chart (Fig. 5C) further revealed that XX wine exhibited the highest sourness and bitterness, whereas HY wines showed more pronounced sweetness. Among the fermented fruit states, DPL fermented wines exhibited the highest bitterness, consistent with their elevated total phenolic and flavonoid content, which contributed to the enhanced bitter sensations. Phenolic compounds contribute to bitterness by interacting with bitter taste receptors on the tongue, while their ability to bind salivary proteins induces astringency through the formation of insoluble protein and polyphenol complexes (Jagatić Korenika et al., 2023). Interestingly, DPL also elevated perceived sourness despite having lower total acid content (Fig. 2B). This discrepancy may be related to acid dissociation or interactions among matrix components (Da Conceicao Neta et al., 2007; Pascual et al., 2016). Thus, total acid content alone may not fully explain the observed sourness differences.

Sensory evaluation further supported the E-tongue results. As shown in Fig. 5D, XX wine showed a more pronounced herbaceous aroma, together with stronger sourness and bitterness, whereas HY and JY wines exhibited more evident fruity and floral attributes.

### 3.5 Relationship among pectin degradation, pectinolytic enzyme activities, and methanol accumulation

#### 3.5.1 Methanol content and net changes of pectin fractions

Methanol, the primary hazardous byproduct in fruit wine, mainly originates from the hydrolysis of pectin methoxyl groups (Shen et al., 2024). OIV specifies methanol limits of 400 mg/L for red wines and 250 mg/L for white and rosé wines (OIV, 2004). These limits for grape wine were used as contextual benchmarks for discussing methanol concentrations in kiwi wine. All reported concentrations were below these reference values. As shown in Fig. 6A, methanol content varied markedly among cultivars: HY wine generally exhibited the lowest levels (96.57–156.33 mg/L), JY wine the highest (142.71–215.03 mg/L), and XX wine intermediate values (129.89–132.41 mg/L). These differences may partly reflect variation in initial pectin content, endogenous PME activity; however, these initial characteristics were not measured, and this explanation remains hypothetical (Ramos-Pineda et al., 2020).

**Fig. 6:**
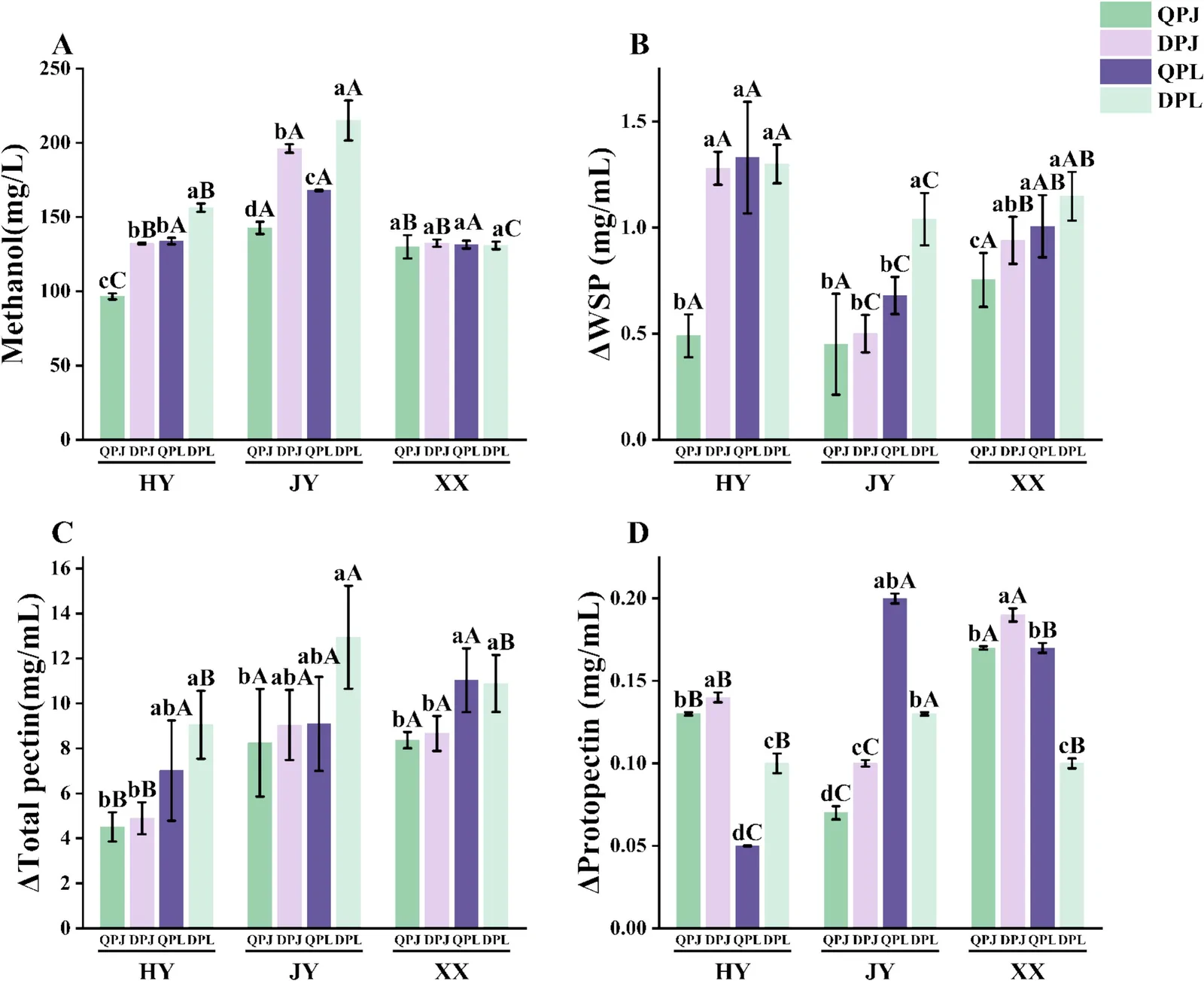
Methanol content and changes in pectin fractions during fermentation: (A) methanol content (mg/L); (B) Water-soluble pectin (WSP) consumption (mg/mL); (C) total pectin consumption (mg/mL); (D) protopectin consumption (mg/mL). Different lowercase letters indicate significant differences (*p* < 0.05) among fruit states within the same cultivar; different uppercase letters indicate significant differences (*p* < 0.05) among cultivars under the same fruit state. Letters should be interpreted only within their respective comparison sets.

Across fermented fruit states, methanol levels were lowest in peeled juice fermentation and increased with peel involvement and pulp maceration in most cases. This pattern suggests that excluding peel and pulp tissues reduced the availability of pectin-rich substrates, thereby limiting methanol formation (Han et al., 2022). The net changes in pectin fractions between day_0_ and day_8_ further supported this interpretation. Water-soluble pectin (WSP) is a readily accessible substrate for PME-mediated demethylation and is directly linked to methanol release (Szatanik-Kloc et al., 2017). In the present study, WSP consumption was generally greater in pulp-fermented samples than in juice-fermented samples, and peel-involved fermentations showed higher WSP depletion than their peeled counterparts (Fig. 6B). These results indicate that pulp contact may enhance the release and utilization of soluble pectin substrates, thereby contributing to higher methanol production. However, these relationships should be interpreted as endpoint net changes rather than dynamic mechanisms during fermentation because pectin fractions were measured only at day_0_ and day_8_. Total pectin consumption showed a similar trend, with DPL treatments exhibiting the greatest overall pectin loss (Fig. 6C), suggesting that combined peel and pulp contact promoted cell wall disassembly and expanded the pectin pool available for enzymatic attack (Romero-Cascales et al., 2012). In contrast, protopectin consumption showed a less consistent relationship with methanol accumulation (Fig. 6D), likely because protopectin must first be converted into soluble pectin before PME-mediated demethylation proceeds (Chandel et al., 2022).

#### 3.5.2 Apparent PG, PL, and PME activities

To characterize variation in enzymatic activity at the fermentation endpoint, the apparent activities of PG, PL, and PME were measured. The commercial pectinase may have contained residual PME side activity, which could have contributed to methanol release and the apparent PME activity. Because all treatments received the same pectinase pretreatment, the observed differences remain interpretable as combined responses under these conditions. PG and PL primarily cleave the pectin backbone, promoting cell wall disassembly and generating oligogalacturonides (Shi et al., 2022; Yang et al., 2021). As shown in Fig. 7A and B, PG and PL activities were generally higher in QPJ than in DPJ treatments, indicating that peel-derived components may exert an inhibitory effect on these backbone-cleaving enzymes (Campos et al., 2025). PME is the key enzyme directly responsible for methanol formation, catalyzing the removal of methyl ester groups from pectin and releasing methanol as a byproduct while producing de-esterified pectin (Frommhagen et al., 2025). As shown in Fig. 7C, PME activity varied markedly among cultivars: JY wines consistently showed the highest activity (22.67–31.33 μmol/h/mL), followed by XX (21.33–28.67 μmol/h/mL) and HY (17.33–27.33 μmol/h/mL). This overall pattern was broadly consistent with the methanol concentrations. Pulp-fermented wines showed higher endpoint PME activity than juice-fermented wines, alongside higher methanol concentrations.

**Fig. 7:**
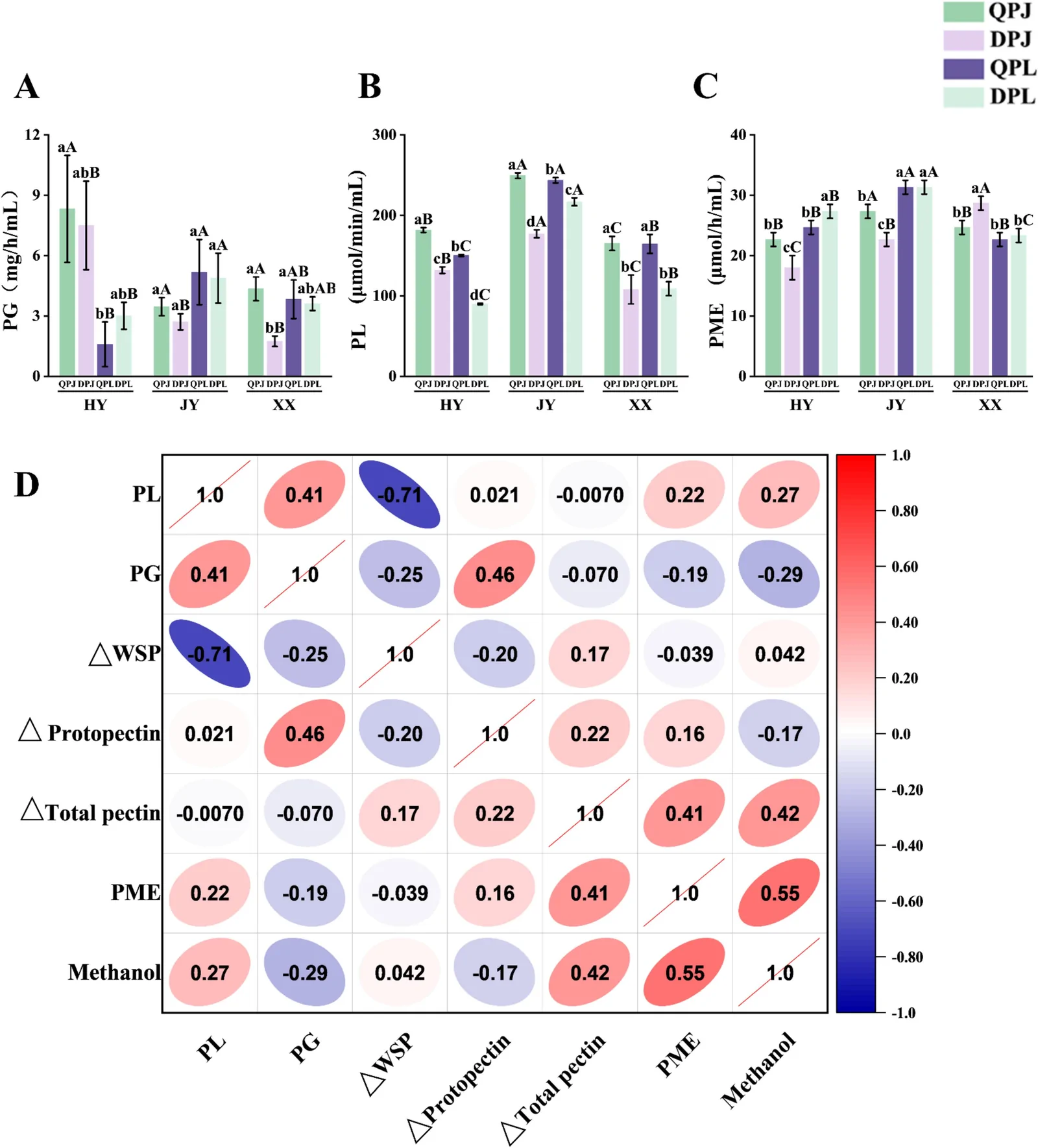
Apparent pectinolytic enzyme activities and correlation analysis: (A) apparent polygalacturonase (PG) activity (mg/h/mL); (B) apparent pectinlyase activity (PL) (μmol/min/mL); (C) apparent pectin methylesterase activity (PME) (μmol/h/mL); (D) Pearson correlation matrix among pectinolytic enzyme activities, pectin fraction changes, and methanol content. Different lowercase letters indicate significant differences (*p* < 0.05) among fruit states within the same cultivar; different uppercase letters indicate significant differences (*p* < 0.05) among cultivars under the same fruit state. Letters should be interpreted only within their respective comparison sets.

However, endpoint enzyme measurements do not establish the extent of pectin backbone degradation or the relative contributions of different enzymes to methanol formation during fermentation. The higher endpoint methanol concentrations observed in pulp-fermented samples may be related to two complementary processes proposed in the literature: (i) The physical disruption of peel and pulp during maceration releases pectin-rich substrates into the fermenting matrix (Miljić et al., 2016); and (ii) prolonged contact between these substrates and pectinolytic enzymes promotes demethylation and methanol release (Han et al., 2022).

#### 3.5.3 Pearson correlation and PLS regression analyses

Pearson correlation analysis was conducted to further clarify the relationships among enzyme activities, pectin fraction changes, and methanol accumulation (Fig. 7D). PL activity was positively correlated with PG activity (*r* = 0.41), suggesting that these two enzymes may act together at endpoint in concert during pectin backbone cleavage and cell wall disassembly, collectively influencing the accessibility of pectin substrates for downstream demethylation. This cooperative relationship is consistent with previous studies suggesting that PG and PL promote pectin depolymerization and act synergistically with PME, thereby facilitating methanol formation (Irshad et al., 2017). Notably, PL activity was negatively correlated with WSP consumption (*r* = −0.71), the strongest association observed in the matrix. This may reflect the ability of PL to cleave insoluble cell wall pectin and release soluble fragments, effectively replenishing the WSP pool and sustaining its turnover rather than depleting it (Liu et al., 2025). PG activity was positively associated with protopectin consumption (*r* = 0.46), implying that increased PG activity may reflect more active degradation or conversion of insoluble pectin fractions (Williams et al., 2001).

Total pectin consumption was positively correlated with PME activity (*r* = 0.41), consistent with the view that overall pectin degradation is accompanied by PME-mediated demethoxylation (Jermendi et al., 2022). Methanol content was moderately positively correlated with PME activity (*n* = 36, *r* = 0.55, R^2^ = 0.30, *p* < 0.001, 95% CI: 0.27–0.74), suggesting that PME may contribute to methanol accumulation in kiwi wine. To further evaluate the relative importance, a PLS regression model was established using methanol concentration as the response variable. The PLS model yielded R^2^Y = 0.503 and Q^2^ = 0.396. Total pectin consumption had the highest VIP score, followed by PME activity (Fig. S3). Among the three pectinolytic enzymes, PME showed the greatest relative importance (VIP = 1.18). These results suggest that PME is a major contributing enzymatic factor associated with methanol formation. By contrast, PG and PL showed weaker and non-significant associations with methanol, indicating that their contribution is indirect — primarily through promoting substrate accessibility for PME — rather than through direct methanol release (Shi et al., 2022). Collectively, these results suggest that methanol formation in kiwi wine is governed principally by the overall pectin degradation and PME-mediated demethoxylation, rather than by changes in any single pectin fraction.

### 3.6 Integrated evaluation of flavor quality and methanol control

The presence of peel and pulp solids elevated the OAV of several ester compounds, thereby intensifying fruity and floral aroma characteristics. However, extensive maceration also facilitated the release of polyphenols and flavonoids, which may impart stronger bitterness. In addition, peel and pulp contact was associated with higher methanol accumulation, highlighting the need to balance aroma enrichment against methanol risk in kiwi wine production.

To integrate these competing factors, entropy-weighted TOPSIS was employed to combine total OAV, sensory fruity/floral intensity, bitterness, and methanol concentration into a single quantitative score (Table S9). Among the three cultivars, HY wine maintained comparatively low methanol levels while retaining a fruity flavor even under DPL conditions with the highest composite score (C = 0.6836), suggesting that HY-DPL merits further evaluation as a candidate production strategy, with sensory quality and methanol risk assessed together. JY wines showed the highest methanol levels, with JY-DPL reaching 215.03 mg/L, close to the 250 mg/L OIV benchmark. In the TOPSIS ranking, JY-QPL (C = 0.6254) and JY-QPJ (C = 0.6167) ranked highest within this cultivar, favoring peeled treatments. Juice fermentation may be preferred when methanol reduction is prioritized. For XX wine, the high TPC and TFC values associated with maceration may intensify bitterness, and sensory balance should therefore be prioritized when considering extended peel and pulp contact (XX-DPL, C = 0.5650). Overall, the differential responses of flavor quality and methanol accumulation among cultivars underscore the need for tailored, cultivar-specific fermentation strategies.

## 4 Conclusion

This study demonstrated that the fermented fruit state is a key determinant of flavor quality and methanol formation in kiwi wine, with its effects modulated by cultivar backgrounds. Pulp maceration generally increased phenolic and flavonoid contents, whereas the effects of peel involvement on physicochemical and aroma characteristics varied among cultivars. However, these gains were accompanied by increased bitterness and sourness perception, as well as methanol accumulation. The latter was linked to greater WSP consumption, higher total pectin degradation, and elevated apparent PME activity at endpoint. Endpoint PME activity was identified as a contributing enzymatic factor associated with methanol formation. Entropy-weighted TOPSIS ranked HY-DPL highest under the selected criteria, supporting cultivar-specific selection of fermentation conditions to balance aroma, bitterness, and methanol concentration. Future studies should monitor dynamic changes in pectin fractions, enzyme activities, and methanol during fermentation and distinguish the contributions of endogenous enzymes and commercial pectinase to methanol formation. Organic acids should also be quantified to clarify their relationships with perceived sourness. Overall, this work provides a practical framework for balancing aroma enrichment, taste quality, and methanol control in kiwi wine fermentation under comparable commercial pectinase pretreatment conditions.

## Ethics and consent statement

The study was approved by the Scientific Research Ethics Committee of the College of Biosystems Engineering and Food Science, Zhejiang University (Ethics ID:202518). And the methodology was in accordance with the approved guidelines. All sensory panelists were informed and signed an informed consent form before the experiment.

## CRediT authorship contribution statement

**Jiahui Shao:** Writing – original draft, Visualization, Investigation, Conceptualization. **Shiying Huang:** Methodology, Investigation. **Yuzhe Lou:** Methodology, Investigation. **Shiguo Chen:** Writing – review & editing. **Xingqian Ye:** Writing – review & editing, Supervision, Resources. **Huan Cheng:** Writing – review & editing, Supervision, Project administration, Funding acquisition.

## Declaration of competing interest

The authors declare that they have no known competing financial interests or personal relationships that could have appeared to influence the work reported in this paper.

## Data Availability

Data will be made available on request.
