# Supplementary material for: Fermented fruit state shapes flavor quality and methanol accumulation in kiwi wine: A comparative study of three flesh-colored kiwifruit cultivars
Source: Food Chem X. 2026 Oct 5;39:104583. doi: 10.1016/j.fochx.2026.104583 (PMC13679310; doi:10.1016/j.fochx.2026.104583)
Supplement: Supplementary file 1 [file mmc1.docx]

**The following are the Supplementary data to this article:**

Table S1. The physicochemical characteristics for kiwifruit.

| **Cultivar** | **Initial TSS (ºBrix)** | **Total acidity (g/L)** | **pH** |
| --- | --- | --- | --- |
| Hongyang | 16 | 13.95 ± 1.45 | 3.56 ± 0.21 |
| Jinyan | 15 | 14.86 ± 0.56 | 3.48 ± 0.43 |
| Xuxiang | 15 | 15.41 ± 0.84 | 3.61 ± 0.55 |

Table S2. Validation parameters for methanol determination in kiwi wine by GC–FID.

| **Compound** | **Linearity** | | | **LOD**  **(mg/L)** | **LOQ**  **(mg/L)** | **Intraday precision (%)** | **Interday precision (%)** | **Recovery rate (%)** |
| --- | --- | --- | --- | --- | --- | --- | --- | --- |
|  | **Slope** | **Intercept** | **R^2^** |  |  |  |  |  |
| Methanol | 0.0024 | -0.0761 | 0.9976 | 7.65 | 24.32 | 2.88 | 3.04 | 93.32 |

Note: Calibration was performed by plotting the methanol to internal standard peak area ratio against the corresponding concentration ratio over a methanol concentration range of 0-400 mg/L. LOD and LOQ were estimated from methanol standards analyzed under the same GC–FID conditions at S/N of approximately 3 and 10, respectively, following Wang et al. (2022). Intraday and interday precision are expressed as relative standard deviations (RSDs), based on 6 replicate measurements within one day and 6 measurements per day over 3 days, respectively. Recovery was assessed by spiking kiwi wine with methanol at 0.05, 0.10, and 0.15 mg/mL before distillation. Recovery was calculated as:

$$R(\%)=\frac{C_{\mathrm{spiked}}-C_{\mathrm{unspiked}}}{C_{\mathrm{added}}}\times100$$

Table S3. Two-way ANOVA interaction effects.

| **Index** | **cultivar × fruit state F; *p*** | **peel × pulp F; *p*** |
| --- | --- | --- |
| pH | 20.71; 2.13×10⁻⁸ | 21.89; 9.39×10⁻⁵ |
| Total acid | 1.57; 0.200 | 0.08; 0.777 |
| Reducing sugar | 6.74; 0.000278 | 7.06; 0.0138 |
| Alcohol content | 11.94; 3.36×10⁻⁶ | 1.54; 0.227 |
| TSS | 24.63; 3.83×10⁻⁹ | 0.73; 0.401 |
| Sugar-acid ratio | 5.70; 0.000848 | 7.61; 0.0109 |
| Total phenolic content | 1.97; 0.110 | 5.46; 0.0281 |
| Total flavonoid content | 9.78; 1.74×10⁻⁵ | 0.98; 0.333 |
| Total pectin | 0.92; 0.498 | 1.81; 0.191 |
| Protopectin | 718.90; 7.42×10⁻²⁶ | 519.73; 9.00×10⁻¹⁸ |
| Water-soluble pectin | 5.31; 0.00131 | 3.84; 0.0618 |
| Methanol | 33.26; 1.73×10⁻¹⁰ | 4.88; 0.0370 |
| PME activity | 28.76; 7.86×10⁻¹⁰ | 12.07; 0.00196 |
| PL activity | 16.63; 1.74×10⁻⁷ | 5.47; 0.0280 |
| PG activity | 3.76; 0.00885 | 1.40; 0.249 |

Table S4. Indicators and weights for evaluating the flavor-methanol trade-off in kiwi wine.

| **Number** | **Index** | **Meaning** | **Index type** | **Weight** |
| --- | --- | --- | --- | --- |
| 1 | Total OAV | Representing aroma potential. | Benefit indicator | 0.1966 |
| 2 | Sensory fruity intensity | Represents perceived fruity aroma intensity. | Benefit indicator | 0.2339 |
| 3 | Sensory floral intensity | Represents perceived floral aroma intensity. | Benefit indicator | 0.2256 |
| 4 | Sensory bitterness intensity | Represents perceived bitterness. | Cost indicator | 0.2096 |
| 5 | Methanol concentration | Represents measured methanol concentration. | Cost indicator | 0.1343 |

**Note:** Entropy weighting assigned weights according to variation among treatments, reducing reliance on subjective weight assignment.

Table S5. Semi-quantitative concentrations of volatile compounds in the 12 kiwi wines determined by GC-MS (μg/L).

| **Volatile compounds** | **HY-QPJ** | **HY-DPJ** | **HY-QPL** | **HY-DPL** | **JY-QPJ** | **JY-DPJ** | **JY-QPL** | **JY-DPL** | **XX-QPJ** | **XX-DPJ** | **XX-QPL** | **XX-DPL** |
| --- | --- | --- | --- | --- | --- | --- | --- | --- | --- | --- | --- | --- |
| **Acid** |  |  |  |  |  |  |  |  |  |  |  |  |
| Acetic acid | 363.58 ± 55.71 | 68.12 ± 12.43 | 403.30 ± 183.27 | 750.91 ± 61.70 | 947.85 ± 403.58 | 94.17 ± 2.55 | 698.57 ± 172.56 | 132.88 ± 41.55 | 222.98 ± 61.62 | 139.62 ± 18.15 | 365.23 ± 68.07 | 260.15 ± 22.13 |
| Isobutyric acid | 25.86 ± 3.69 | 5.18 ± 0.87 | 20.85 ± 7.28 | 29.69 ± 3.37 | 82.27 ± 26.22 | 3.38 ± 0.93 | 70.86 ± 14.87 | - | 39.46 ± 4.78 | 29.46 ± 2.33 | 75.73 ± 8.07 | 38.65 ± 2.07 |
| Hexanoic acid | 47.03 ± 0.64 | 39.33 ± 14.80 | - | 206.40 ± 13.63 | 115.45 ± 44.77 | - | 109.29 ± 14.17 | - | - | 136.29 ± 17.34 | 273.08 ± 44.63 | 202.49 ± 12.71 |
| Octanoic acid | 289.13 ± 164.30 | 147.84 ± 32.38 | 135.66 ± 55.95 | 295.68 ± 45.34 | 122.61 ± 33.49 | 66.03 ± 17.01 | 123.41 ± 13.41 | 47.01 ± 9.58 | 164.76 ± 6.19 | 381.32 ± 95.41 | 621.01 ± 115.06 | - |
| Decanoic acid | 83.58 ± 46.20 | 32.05 ± 8.96 | 10.22 ± 3.44 | 45.29 ± 3.36 | 33.49 ± 19.67 | 6.60 ± 0.82 | 36.57 ± 19.02 | 10.33 ± 5.65 | 46.22 ± 7.28 | 169.06 ± 17.85 | 86.30 ± 18.57 | - |
| Butyric Acid | - | 2.42 ± 0.78 | 7.29 ± 3.14 | 15.56 ± 1.52 | - | - | 10.62 ± 1.77 | 3.46 ± 0.67 | - | - | 60.92 ± 8.33 | - |
| TRANS-2-HEXENOIC ACID | - | - | - | - | - | - | - | - | - | 10.10 ± 0.68 | - | - |
| Propionic acid | - | - | 9.57 ± 4.78 | 13.29 ± 0.98 | 14.69 ± 8.95 | - | 8.68 ± 1.88 | - | - | - | - | - |
| 2-Methyl butyric acid | - | - | - | - | - | - | 39.08 ± 12.43 | - | - | - | - | - |
| Isovaleric acid | - | 9.18 ± 0.77 | 25.52 ± 9.66 | 27.45 ± 5.58 | - | - | - | - | - | - | - | - |
| Benzoic acid | - | 1.48 ± 0.55 | - | 3.15 ± 0.72 | - | - | - | - | - | - | - | - |
| Lauric acid | - | 1.98 ± 0.97 | - | 5.21 ± 1.35 | - | - | - | - | - | - | - | - |
| Glyoxylic acid | - | 2.70 ± 1.26 | - | - | - | - | - | - | - | - | - | - |
|  |  |  |  |  |  |  |  |  |  |  |  |  |
| **Alcohol** |  |  |  |  |  |  |  |  |  |  |  |  |
| 1-Propanol | 1325.53 ± 304.24 | 650.95 ± 222.12 | 2080.36 ± 1120.67 | 2595.05 ± 236.67 | 1514.42 ± 493.89 | 265.89 ± 71.63 | 1567.70 ± 357.61 | 259.06 ± 46.11 | 314.94 ± 89.36 | 185.56 ± 5.14 | 271.13 ± 52.06 | 230.05 ± 23.82 |
| 3-METHYL-2-BUTANOL | - | - | 5.79 ± 0.51 | - | - | - | - | - | - | 4.81 ± 2.06 | - | 18.23 ± 4.09 |
| 2-Methyl-1-propanol | 2305.54 ± 213.78 | 741.56 ± 251.57 | 1604.30 ± 729.52 | 1750.17 ± 153.65 | 4998.24 ± 1666.19 | 684.64 ± 207.93 | 3896.84 ± 662.79 | 464.41 ± 57.80 | 4972.40 ± 1584.45 | 3022.19 ± 185.83 | 5720.93 ± 1032.98 | 3032.05 ± 132.90 |
| 3-Methyl-1-butanol | 6865.84 ± 535.56 | 3557.49 ± 778.35 | 3518.12 ± 807.69 | 11173.53 ± 1693.91 | 13606.77 ± 3003.65 | 4595.33 ± 960.46 | 11742.40 ± 628.49 | 3434.99 ± 537.95 | 11327.22 ± 3647.22 | 9100.78 ± 411.44 | 12856.26 ± 2306.34 | 8964.71 ± 589.70 |
| 1-Pentanol | 12.17 ± 4.37 | 1058.26 ± 24.91 | 16.97 ± 1.99 | 28.45 ± 0.94 | - | - | - | - | - | 10.58 ± 0.82 | 14.11 ± 1.25 | 14.79 ± 2.43 |
| 3-METHYL-1-PENTANOL | - | 2.13 ± 0.61 | - | 9.92 ± 1.23 | - | - | - | - | 7.71 ± 0.72 | 6.23 ± 0.25 | 8.11 ± 2.34 | 6.29 ± 1.92 |
| 1-Hexanol | 29.81 ± 7.60 | 143.89 ± 15.48 | - | 495.77 ± 46.01 | 464.86 ± 164.35 | 362.05 ± 24.22 | 387.20 ± 25.35 | 411.89 ± 51.25 | 359.95 ± 53.10 | 407.86 ± 1.26 | 436.52 ± 33.67 | 500.97 ± 27.44 |
| TRANS-3-HEXEN-1-OL | - | 7.60 ± 2.17 | - | 12.40 ± 1.76 | 84.73 ± 2.89 | - | 18.95 ± 7.19 | - | 25.69 ± 1.66 | - | 29.96 ± 0.04 | 23.35 ± 2.93 |
| 1-Heptanol | - | 3.27 ± 1.10 | 15.37 ± 8.96 | 28.57 ± 3.63 | - | - | 245.22 ± 3.13 | - | - | 10.61 ± 2.26 | 25.08 ± 10.26 | 22.29 ± 7.99 |
| 2-Ethylhexanol | - | - | - | - | - | - | - | - | - | 13.66 ± 1.24 | - | 17.14 ± 3.90 |
| 2,3-Butanediol | 185.98 ± 37.78 | 70.61 ± 15.51 | 117.21 ± 58.98 | 766.17 ± 97.34 | 218.73 ± 95.07 | 28.51 ± 5.27 | 232.85 ± 48.64 | 31.61 ± 16.62 | 59.02 ± 7.93 | 66.43 ± 12.24 | 140.52 ± 27.91 | 102.55 ± 6.38 |
| Linalool | - | - | - | - | 15.94 ± 6.72 | - | 92.22 ± 0.05 | - | - | 99.57 ± 4.64 | - | 143.35 ± 1.10 |
| 1-Octanol | 6.90 ± 0.53 | 5.27 ± 1.73 | 6.05 ± 1.75 | 20.95 ± 2.43 | - | 7.01 ± 2.36 | - | 8.99 ± 2.58 | - | 6.54 ± 1.44 | 10.09 ± 0.22 | 13.39 ± 0.73 |
| (R,R)-2,3-Butanediol | 50.28 ± 14.35 | 17.83 ± 1.85 | 38.37 ± 13.05 | 208.00 ± 36.35 | 64.14 ± 6.63 | - | 60.07 ± 9.44 | - | 45.65 ± 18.92 | 24.55 ± 5.09 | 46.36 ± 5.72 | 33.76 ± 7.36 |
| alpha-Terpineol | - | - | - | - | - | - | - | - | - | - | - | 34.86 ± 6.67 |
| 3-Methylthiopropanol | - | - | - | 6.22 ± 0.52 | 11.84 ± 7.50 | - | 14.88 ± 2.64 | - | 9.94 ± 4.79 | 12.08 ± 3.08 | 9.65 ± 2.06 | 8.18 ± 0.58 |
| Citronellol | 15.73 ± 1.87 | 4.27 ± 1.14 | 3.71 ± 1.02 | 9.36 ± 2.34 | 23.17 ± 2.14 | - | - | - | 23.82 ± 2.21 | 149.92 ± 3.89 | 12.15 ± 2.12 | 94.97 ± 2.87 |
| Geraniol | - | - | - | - | - | - | - | - | - | - | - | 24.54 ± 1.79 |
| Benzyl alcohol | - | - | - | - | - | - | - | - | 10.85 ± 2.92 | 9.79 ± 2.32 | 50.06 ± 10.87 | 61.03 ± 2.84 |
| Phenethyl alcohol | 529.68 ± 189.48 | 229.15 ± 49.80 | 590.76 ± 9.19 | 1408.86 ± 175.72 | 1892.73 ± 474.34 | 269.52 ± 79.18 | 1735.82 ± 863.64 | 384.50 ± 136.80 | 1643.44 ± 612.40 | 1241.75 ± 231.69 | 3.62 ± 1.34 | 1072.78 ± 46.86 |
| 1-Butanol | 18.68 ± 9.21 | 16.48 ± 1.58 | 24.20 ± 15.31 | 35.61 ± 13.67 | - | 14.49 ± 6.27 | 23.04 ± 7.12 | 6.80 ± 1.12 | 14.84 ± 5.15 | 7.91 ± 3.03 | 14.57 ± 1.78 | - |
| CIS-2-PENTEN-1-OL | - | - | - | - | - | - | - | - | 7.35 ± 0.58 | - | - | - |
| 4-Methyl-1-pentanol | - | - | 6.21 ± 0.05 | 6.86 ± 0.04 | - | - | - | - | - | - | 5.68 ± 0.42 | - |
| Leaf alcohol | - | - | - | - | - | - | - | - | - | 30.00 ± 4.29 | - | - |
| NEROL | - | - | - | - | - | - | - | - | - | 10.85 ± 2.15 | - | - |
| 1,8-Cineole | - | - | - | - | 33.90 ± 7.10 | - | 39.03 ± 3.76 | - | - | - | - | - |
| 2-Methyl-1-butanol | - | - | - | - | 1585.16 ± 109.75 | - | - | - | - | - | - | - |
| Terpinen-4-ol | 25.00 ± 8.47 | - | 24.43 ± 4.33 | 24.63 ± 3.90 | 33.67 ± 8.28 | - | 31.23 ± 2.26 | - | - | - | - | - |
| 2-Heptanol | - | - | - | - | - | - | 8.43 ± 2.19 | - | - | - | - | - |
| 5-METHYL-2-HEPTANOL | - | - | - | - | - | - | 16.81 ± 7.44 | - | - | - | - | - |
| Propylene glycol | - | 4.15 ± 2.84 | - | - | - | - | 12.05 ± 5.55 | - | - | - | - | - |
| 1,3-Butanediol | - | - | - | - | - | 21.82 ± 6.07 | - | - | - | - | - | - |
| 1-Nonanol | - | - | 27.05 ± 5.36 | 31.82 ± 3.99 | - | 12.05 ± 2.86 | - | 11.66 ± 2.45 | - | - | - | - |
| 2-Ethyl-1-butanol | - | - | - | 4.42 ± 0.17 | - | - | - | - | - | - | - | - |
| 6-METHYL-2-HEPTANOL | - | - | - | 30.41 ± 12.31 | - | - | - | - | - | - | - | - |
| 2-NONANOL | - | - | 13.08 ± 2.50 | 18.20 ± 2.87 | - | - | - | - | - | - | - | - |
| cis-6-Nonen-1-ol | - | - | - | 14.06 ± 2.23 | - | - | - | - | - | - | - | - |
| Decyl alcohol | - | 1.91 ± 0.50 | - | - | - | - | - | - | - | - | - | - |
| Glycerol | - | 7.46 ± 0.24 | - | 16.32 ± 1.70 | 13.94 ± 3.18 | - | - | - | - | - | - | - |
|  |  |  | - |  |  |  |  |  |  |  |  |  |
| **Aldehyde** |  |  |  |  |  |  |  |  |  |  |  |  |
| 2,4-Dimethylbenzaldehyde | - | - | - | - | - | - | - | - | - | - | 14.29 ± 2.13 | 10.85 ± 0.63 |
| 4-Ethylbenzaldehyde | - | - | - | - | - | 7.54 ± 1.51 | - | 4.62 ± 3.37 | 11.39 ± 3.56 | - | - | - |
| Benzaldehyde | - | - | - | - | - | 8.78 ± 2.34 | - | 15.87 ± 10.93 | - | - | 7.75 ± 1.57 | - |
| 1-Nonanal | - | 7.22 ± 0.06 | - | - | - | - | - | - | - | 16.34 ± 1.21 | - | - |
| Octanal | - | - | 1.32 ± 0.32 | - | - | 3.92 ± 2.39 | - | 4.16 ± 0.15 | - | - | - | - |
|  |  |  | - |  |  |  |  |  |  |  |  |  |
| **Ester** |  |  |  |  |  |  |  |  |  |  |  |  |
| Isobutyl acetate | 81.44 ± 3.29 | 24.57 ± 1.85 | 39.84 ± 6.57 | 129.01 ± 22.62 | 114.05 ± 29.82 | 67.32 ± 7.80 | 137.85 ± 17.96 | 22.21 ± 9.48 | 127.37 ± 13.60 | 92.14 ± 5.60 | 136.92 ± 22.44 | 96.17 ± 4.75 |
| Ethyl butyrate | - | 12.59 ± 1.25 | 32.58 ± 6.71 | 54.65 ± 3.85 | 46.40 ± 16.31 | 208.12 ± 32.49 | 33.98 ± 8.86 | 324.89 ± 27.05 | 58.95 ± 9.08 | 46.18 ± 5.02 | 97.67 ± 13.27 | 73.18 ± 13.34 |
| Isoamyl acetate | 371.78 ± 89.48 | 288.89 ± 157.56 | 456.68 ± 98.88 | 983.97 ± 92.90 | 816.45 ± 320.40 | 921.53 ± 88.70 | 808.94 ± 142.24 | 582.91 ± 34.91 | 979.36 ± 179.47 | 543.61 ± 78.68 | 1337.66 ± 166.60 | 858.67 ± 82.75 |
| Methyl hexanoate | - | - | - | 10.81 ± 4.74 | - | 6.59 ± 2.27 | - | 4.33 ± 0.17 | - | 6.23 ± 0.66 | 9.87 ± 1.43 | 5.35 ± 0.27 |
| Ethyl Hexanoate | 213.31 ± 53.59 | 172.95 ± 39.89 | 155.60 ± 13.68 | 665.39 ± 138.12 | 209.00 ± 55.43 | 954.67 ± 34.01 | 272.43 ± 30.47 | 650.58 ± 25.41 | 481.18 ± 59.78 | 391.58 ± 30.87 | 1101.11 ± 68.62 | 653.18 ± 13.52 |
| Hexyl acetate | - | 16.92 ± 2.90 | - | 147.54 ± 43.36 | 70.21 ± 29.30 | 385.25 ± 15.62 | 77.29 ± 19.32 | 311.90 ± 18.08 | 54.20 ± 4.21 | 56.25 ± 5.43 | 142.97 ± 30.15 | 147.59 ± 35.78 |
| TRANS-3-HEXENYL ACETATE | - | - | - | - | - | - | - | - | - | 4.35 ± 0.21 | 9.09 ± 3.43 | 7.38 ± 2.03 |
| ethyl hex-2-enoate | - | 3.33 ± 1.13 | - | - | 28.21 ± 8.85 | - | - | - | 48.63 ± 4.81 | 26.47 ± 2.11 | 25.34 ± 8.28 | 13.14 ± 1.80 |
| Caprylic acid methyl ester | - | 7.14 ± 3.05 | 10.99 ± 3.03 | 24.41 ± 3.89 | 17.85 ± 5.70 | 35.06 ± 3.49 | 16.05 ± 1.53 | 28.76 ± 1.87 | 16.79 ± 2.84 | 26.26 ± 6.06 | 17.75 ± 1.27 | 46.59 ± 11.26 |
| Ethyl caprylate | - | 1637.63 ± 106.63 | 1908.14 ± 735.74 | 4253.86 ± 476.21 | 2060.07 ± 887.34 | 3062.51 ± 320.33 | 1475.74 ± 714.66 | 3472.89 ± 480.13 | 1061.72 ± 134.77 | 1372.24 ± 147.96 | 2364.51 ± 503.50 | 2104.80 ± 403.67 |
| Ethyl 3-hydroxybutyrate | - | - | 11.09 ± 5.19 | 22.78 ± 14.58 | - | - | - | - | - | 11.47 ± 3.69 | - | 15.38 ± 0.93 |
| Ethyl nonanoate | - | 7.48 ± 0.42 | 11.99 ± 2.89 | 25.29 ± 2.96 | - | 5.13 ± 0.73 | - | 7.41 ± 5.24 | - | - | 8.07 ± 2.84 | 5.36 ± 1.52 |
| Methyl decanoate | - | - | 10.86 ± 5.53 | 17.26 ± 3.81 | - | 8.88 ± 5.25 | 16.29 ± 2.02 | 7.52 ± 4.96 | - | 17.41 ± 7.77 | 13.86 ± 4.86 | 21.81 ± 2.15 |
| Methyl benzoate | - | - | - | - | - | - | - | - | 93.32 ± 28.83 | 56.79 ± 0.13 | - | 73.61 ± 2.86 |
| Ethyl caprate | 719.01 ± 399.16 | 555.11 ± 110.50 | 1626.40 ± 536.25 | 1582.42 ± 221.88 | 2683.38 ± 642.69 | 1248.25 ± 350.44 | 1836.67 ± 346.89 | 1293.64 ± 595.48 | 537.37 ± 28.04 | 523.60 ± 47.51 | - | 636.25 ± 79.23 |
| Ethyl benzoate | - | - | - | - | - | - | - | - | 13.87 ± 0.49 | 25.57 ± 0.45 | 37.90 ± 3.74 | 34.02 ± 0.38 |
| Benzyl acetate | - | - | - | - | - | - | - | - | - | - | - | 7.98 ± 0.12 |
| Methyl salicylate | - | - | - | - | 43.56 ± 12.68 | - | 56.09 ± 2.51 | - | - | - | 8.48 ± 0.71 | 17.77 ± 1.01 |
| Phenethyl acetate | 110.61 ± 27.49 | 50.04 ± 9.07 | 122.73 ± 13.02 | 224.43 ± 18.72 | 166.52 ± 41.71 | 56.29 ± 10.39 | 224.14 ± 37.10 | 46.32 ± 3.82 | 189.46 ± 28.29 | 176.09 ± 10.88 | 202.45 ± 17.46 | 148.80 ± 6.65 |
| N-CAPRIC ACID ISOAMYL ESTER | 17.36 ± 0.35 | 2.78 ± 1.54 | 9.20 ± 2.32 | 7.90 ± 1.63 | 18.47 ± 7.32 | - | 21.54 ± 1.77 | - | - | 5.05 ± 0.61 | - | 6.38 ± 1.55 |
| Ethyl myristate | 7.29 ± 0.15 | 2.94 ± 1.53 | 4.45 ± 1.45 | 12.82 ± 2.16 | 10.92 ± 1.77 | - | 9.11 ± 1.56 | - | - | - | 12.80 ± 1.81 | 10.02 ± 3.27 |
| Palmitic acid ethyl ester | 10.86 ± 3.90 | 5.85 ± 1.02 | 16.69 ± 4.37 | 41.19 ± 1.58 | 36.04 ± 12.12 | - | 30.42 ± 3.18 | 4.06 ± 2.24 | 6.74 ± 1.36 | 8.40 ± 1.53 | 54.04 ± 20.43 | 21.27 ± 8.27 |
| N-CAPRYLIC ACID ISOBUTYL ESTER | - | - | - | - | - | - | 17.59 ± 9.18 | - | 22.92 ± 1.23 | - | 37.73 ± 3.29 | - |
| Isobutyl hexanoate | - | - | - | - | - | - | - | - | - | - | 9.57 ± 3.73 | - |
| PENTYL HEXANOATE | - | - | - | - | - | - | - | - | - | - | 23.91 ± 9.75 | - |
| ISOAMYL HEXANOATE | - | - | - | - | - | - | - | - | - | - | 15.61 ± 1.67 | - |
| Diethyl succinate | 9.57 ± 0.64 | 1.49 ± 0.29 | 8.59 ± 0.63 | 12.46 ± 1.04 | - | - | 10.80 ± 1.77 | - | - | - | 22.99 ± 2.99 | - |
| ISOBUTYL DECANOATE | - | - | 6.60 ± 2.05 | - | - | - | - | - | - | - | 7.04 ± 0.78 | - |
| CENTIFOLYL | 161.66 ± 5.90 | - | - | - | - | - | - | - | - | 12.66 ± 3.70 | - | - |
| Diethyl sulfite | - | - | - | - | 9.78 ± 4.31 | - | - | - | - | - | - | - |
| Ethyl laurate | 227.77 ± 133.76 | 185.88 ± 62.74 | 235.40 ± 100.06 | 184.47 ± 61.30 | 314.12 ± 50.07 | 227.62 ± 131.52 | 386.01 ± 135.79 | 328.11 ± 41.76 | - | - | - | - |
| FEMA 2080 | 4.63 ± 0.43 | 6.06 ± 1.86 | 25.41 ± 9.19 | 25.02 ± 0.02 | 23.98 ± 2.21 | - | 27.26 ± 4.16 | - | - | - | - | - |
| cis-3-Hexenyl Acetate | - | - | - | - | - | 5.99 ± 2.44 | - | 139.13 ± 28.02 | - | - | - | - |
| Ethyl 2-furoate | - | 4.27 ± 0.79 | - | 983.97 ± 92.90 | - | - | - | 5.01 ± 2.99 | - | - | - | - |
| Ethyl heptanoate | - | - | - | 9.01 ± 2.08 | - | - | - | - | - | - | - | - |
| PROPYL CAPRYLATE | - | - | 10.11 ± 6.01 | 13.44 ± 3.07 | - | - | - | - | - | - | - | - |
| OCTYL FORMATE | - | - | - | 20.27 ± 3.00 | - | - | - | - | - | - | - | - |
| 3-Hydroxyhexanoic Acid Methyl Ester | - | - | 8.98 ± 2.57 | 21.93 ± 1.61 | - | - | - | - | - | - | - | - |
| Dimethyl phthalate | - | - | - | 7.28 ± 1.51 | - | - | - | - | - | - | - | - |
| N-PROPYL DECANOATE | - | - | 9.62 ± 5.78 | 12.70 ± 4.32 | - | - | - | - | - | - | - | - |
| N-NITROSO-N-METHYLURETHANE | - | 0.77 ± 0.36 | - | - | - | - | - | - | - | - | - | - |
| 4-Methyl-2-(2-methyl-1-propenyl) | - | - | - | - | - | - | - | - | - | 19.35 ± 0.87 | - | 42.16 ± 0.79 |
|  |  |  | - |  |  |  |  |  |  |  |  |  |
| **Ketone** |  |  |  |  |  |  |  |  |  |  |  |  |
| 2-Octanone | 6.47 ± 0.69 | 4.70 ± 0.60 | 12.70 ± 7.36 | 16.03 ± 4.67 | - | - | - | - | - | - | 14.56 ± 0.65 | - |
| Acetoin | 69.88 ± 17.22 | 4.95 ± 2.54 | 78.34 ± 30.89 | 208.43 ± 13.63 | 96.85 ± 31.70 | - | 145.40 ± 31.77 | - | - | - | - | - |
| 4-Hydroxy-2-butanone | - | 4.38 ± 1.24 | - | - | 24.29 ± 12.25 | - | - | - | - | - | - | - |
| Dihydro-2-methyl-3(2H)-thiophenone | - | 37.27 ± 2.40 | - | - | - | - | - | - | - | - | - | - |
| D-CAMPHOR | - | - | - | - | 15.67 ± 4.99 | - | - | - | - | - | - | - |
| Camphor | - | - | - | - | - | - | 22.64 ± 2.64 | - | - | - | - | - |
|  |  |  | - |  |  |  |  |  |  |  |  |  |
| **Phenolic** |  |  |  |  |  |  |  |  |  |  |  |  |
| 4-Hydroxy-3-methoxystyrene | 50.44 ± 4.23 | 52.26 ± 13.52 | 23.26 ± 7.41 | 257.50 ± 20.51 | 39.14 ± 10.90 | - | 62.65 ± 32.89 | 5.49 ± 1.53 | 15.09 ± 2.87 | 71.11 ± 15.98 | 169.13 ± 39.52 | 158.82 ± 41.25 |
| 2,4-Di-tert-butylphenol | 22.51 ± 6.81 | 5.17 ± 2.82 | 3.11 ± 0.41 | 9.02 ± 3.99 | - | - | 6.56 ± 0.43 | - | - | - | 5.01 ± 1.02 | - |
| 3,5-DI-TERT-BUTYLPHENOL | - | - | - | - | - | - | - | - | - | 9.18 ± 4.97 | - | - |
| 4-Ethyl-2-methoxyphenol | - | - | - | - | 11.21 ± 1.36 | - | 11.41 ± 3.32 | - | - | - | - | - |
|  |  |  | - |  |  |  |  |  |  |  |  |  |
| **Amides** |  |  | - |  |  |  |  |  |  |  |  |  |
| Hexylamine | - | - | - | - | - | 5.39 ± 1.77 | - | - | 5.11 ± 0.64 | - | - | - |
| Dodecylamine | - | - | 1.28 ± 0.20 | 2.26 ± 0.25 | - | 2.45 ± 0.91 | 13.12 ± 2.16 | 5.32 ± 0.66 | 4.82 ± 0.19 | - | 4.87 ± 2.45 | - |
| Decylamine | - | - | 8.32 ± 3.41 | 11.07 ± 4.00 | - | - | 6.64 ± 0.62 | - | - | - | - | - |
| 1-AMINOHEPTANE | - | - | - | - | - | 5.49 ± 1.85 | - | - | - | - | - | - |
| Octadecanamine | - | - | 1.09 ± 0.52 | 3.17 ± 1.44 | - | - | - | - | - | - | - | - |
|  |  |  |  |  |  |  |  |  |  |  |  |  |
| **Other** |  |  | - |  |  |  |  |  |  |  |  |  |
| N-(3-METHYLBUTYL)ACETAMIDE | - | - | - | - | - | - | - | - | - | 2.72 ± 0.27 | - | - |
| CIS-3-DODECENE | - | - | - | - | - | 27.57 ± 2.82 | - | - | - | - | - | - |
| 2-Methyl-m-dioxane | - | - | - | 76.21 ± 11.62 | - | - | - | - | - | - | - | - |
| Phenylpentane | - | - | - | - | - | 5.64 ± 1.26 | - | - | 12.86 ± 4.03 | 7.78 ± 1.93 | 7.75 ± 1.11 | 8.65 ± 1.38 |
| 3-Ethoxy-1-propanol | 74.73 ± 12.60 | 16.35 ± 2.43 | 26.92 ± 10.25 | 106.23 ± 23.20 | 48.13 ± 10.00 | - | 71.51 ± 24.46 | - | 23.72 ± 3.25 | 7.30 ± 0.25 | 10.34 ± 1.85 | 7.54 ± 1.56 |
|  |  |  | - |  |  |  |  |  |  |  |  |  |
| **Aromatic hydrocarbons** |  |  | - |  |  |  |  |  |  |  |  |  |
| O-CYMENE | - | - | - | - | - | - | - | 6.58 ± 2.48 | - | - | - | - |
| M-CYMENE | - | - | - | - | - | - | - | 11.57 ± 2.31 | - | 17.29 ± 0.65 | - | - |
| 1,3-DI-TERT-BUTYLBENZENE | - | 2.93 ± 0.20 | - | - | - | - | - | - | - | - | - | - |
| o-Xylene | - | - | - | - | - | - | - | - | 15.71 ± 1.26 | 14.15 ± 0.94 | 14.96 ± 3.60 | 15.08 ± 2.27 |
| p-Cymene | - | - | 27.29 ± 9.60 | - | - | 13.12 ± 0.93 | 20.58 ± 11.45 | 250.28 ± 14.30 | - | - | - | 14.13 ± 0.96 |
| **Terpenes** |  |  |  | 0 |  |  |  |  |  |  |  |  |
| (+)-Dipentene | - | - | - | - | - | 16.07 ± 3.32 | 10.71 ± 2.24 | - | 13.75±2.1 | - | 47.78±5.12 | 13.75±2.10 |
| Styrene | 34.5 ± 9.69 | 15.49 ± 1.1 | 51.57 ± 28.43 | 143.02 ± 72.95 | 21.91 ± 1.14 | 54.26 ± 2.18 | 21.15 ± 3.81 | 21.28 ± 9.26 | 26.59±14.08 | 23.1±9.37 | 41.12±16.40 | 26.59±14.08 |
| α-Farnesene | - | - | - | - | - | - | - | - | - | 47.10 ± 0.30 | - | - |
| OCIMENE | - | - | - | - | - | - | - | - | 13.23 ± 2.17 | - | - | 13.23 ± 2.17 |
| Terpinolene | - | - | 14.59 ± 9.16 | - | 25.14 ± 7.12 | - | 28.62 ± 3.13 | - | - | - | - | - |
| Myrcene | - | - | - | - | - | - | - | - | 8.98 ± 2.48 | - | 13.31 ± 2.09 | 8.98 ± 2.48 |

Note: “-” indicates that no qualifying chromatographic peak was detected under the analytical conditions, rather than a concentration below a validated compound-specific LOD.

Table S6. Semi-quantitative estimates of volatile compound class concentrations in kiwi wines (μg/L).

| **Sample** | **Acids** | **Alcohols** | **Esters** | **Other compounds** | **Terpenes** |
| --- | --- | --- | --- | --- | --- |
| HY-QPJ | 809.18 | 11371.14 | 1935.29 | 224.03 | 34.50 |
| HY-DPJ | 310.28 | 6522.28 | 2986.69 | 135.23 | 15.49 |
| HY-QPL | 612.41 | 8091.98 | 4721.95 | 183.63 | 66.16 |
| HY-DPL | 1392.63 | 18695.75 | 9494.28 | 689.92 | 143.02 |
| JY-QPJ | 1316.36 | 24562.24 | 6669.01 | 235.29 | 47.05 |
| JY-DPJ | 170.18 | 6261.31 | 7193.21 | 79.90 | 70.33 |
| JY-QPL | 1097.08 | 20124.74 | 5458.20 | 360.51 | 60.48 |
| JY-DPL | 193.68 | 5013.91 | 7229.67 | 303.89 | 21.28 |
| XX-QPJ | 473.42 | 18822.82 | 3691.88 | 88.70 | 62.55 |
| XX-DPJ | 865.85 | 14421.67 | 3421.70 | 145.87 | 70.20 |
| XX-QPL | 1482.27 | 19654.80 | 5697.34 | 248.66 | 102.21 |
| XX-DPL | 501.29 | 14419.28 | 5046.86 | 215.07 | 62.55 |

Note: Values are semi-quantitative estimates obtained using 2-octanol as the internal standard.

**Table S7.** Active compounds (OAV > 1) in all kiwi wine samples.

| **Volatile compounds** | **Threshold (μg/L)** | **HY-QPJ** | **HY-DPJ** | **HY-QPL** | **HY-DPL** | **JY-QPJ** | **JY-DPJ** | **JY-QPL** | **JY-DPL** | **XX-QPJ** | **XX-DPJ** | **XX-QPL** | **XX-DPL** |
| --- | --- | --- | --- | --- | --- | --- | --- | --- | --- | --- | --- | --- | --- |
| 3-Methyl-1-butanol | 300^b^ | 22.89 | 11.86 | 11.73 | 37.25 | 45.36 | 15.32 | 39.14 | 11.45 | 37.76 | 30.34 | 42.85 | 29.88 |
| 1-Heptanol | 200^a^ | 0.00 | 0.02 | 0.08 | 0.14 | 0.00 | 0.00 | 1.23 | 0.00 | 0.00 | 0.05 | 0.13 | 0.11 |
| Linalool | 3.8^a^ | 0.00 | 0.00 | 0.00 | 0.00 | 4.19 | 0.00 | 24.27 | 0.00 | 0.00 | 26.20 | 0.00 | 37.72 |
| 3-Methylthiopropanol | 5^a^ | 0.00 | 0.00 | 0.00 | 1.24 | 2.37 | 0.00 | 2.98 | 0.00 | 1.99 | 2.42 | 1.93 | 1.64 |
| Citronellol | 10^a^ | 1.57 | 0.43 | 0.37 | 0.94 | 2.32 | 0.00 | 0.00 | 0.00 | 2.38 | 14.99 | 1.22 | 9.50 |
| Phenethyl alcohol | 122^a^ | 4.34 | 1.88 | 4.84 | 11.55 | 15.51 | 2.21 | 14.23 | 3.15 | 13.47 | 10.18 | 0.03 | 8.79 |
| Isoamyl acetate | 5^a^ | 74.36 | 57.78 | 91.34 | 196.79 | 163.29 | 184.31 | 161.79 | 116.58 | 195.87 | 108.72 | 267.53 | 171.73 |
| Methyl decanoate | 6.55^a^ | 0.00 | 0.00 | 1.66 | 2.64 | 0.00 | 1.36 | 2.49 | 1.15 | 0.00 | 2.66 | 2.12 | 3.33 |
| Methyl benzoate | 28^a^ | 0.00 | 0.00 | 0.00 | 0.00 | 0.00 | 0.00 | 0.00 | 0.00 | 3.33 | 2.03 | 0.00 | 2.63 |
| Phenethyl acetate | 20^a^ | 5.53 | 2.50 | 6.14 | 11.22 | 8.33 | 2.81 | 11.21 | 2.32 | 9.47 | 8.80 | 10.12 | 7.44 |
| 4-Hydroxy-3-methoxystyrene | 32^a^ | 1.58 | 1.63 | 0.73 | 8.05 | 1.22 | 0.00 | 1.96 | 0.17 | 0.47 | 2.22 | 5.29 | 4.96 |
| o-cymene | 4.5^a^ | 0.00 | 0.00 | 0.00 | 0.00 | 0.00 | 0.00 | 0.00 | 1.46 | 0.00 | 0.00 | 0.00 | 0.00 |

Note: ^a^ Threshold values determined from literature (Gemert, 2011); ^b^ Threshold values determined from literature (Liu et al., 2014).

Table S8. Tentative identification and responses of selected chromatographic peaks in kiwi wine analyzed by E-nose.

| **Compound** | **RI**  **(MXT-1701)** | **RI (MXT-5)** | **Database RI**  **(MXT-1701/MXT-5)** | **Peak area responses across treatments** | | | | | | | | | | | |
| --- | --- | --- | --- | --- | --- | --- | --- | --- | --- | --- | --- | --- | --- | --- | --- |
|  |  |  |  | **HY-QPJ** | **HY-DPJ** | **HY-QPL** | **HY-DPL** | **JY-QPJ** | **JY-DPJ** | **JY-QPL** | **JY-DPL** | **XX-QPJ** | **XX-DPJ** | **XX-QPL** | **XX-DPL** |
| 1-Propanol | 672 | 531 | 680;534 | 33020.74 ± 775.00 | 31976.50 ± 581.41 | 33689.58 ± 589.93 | 38086.91 ± 1671.21 | 26814.58 ± 598.03 | 29173.02 ± 710.79 | 25035.11 ± 845.64 | 34697.48 ± 355.19 | 13959.75 ± 464.95 | 9582.02 ± 138.22 | 10688.14 ± 436.19 | 8971.27 ± 341.67 |
| 2-Methyl-1-propanol | 738 | 623 | 750;629 | 39503.44 ± 970.46 | 25441.09 ± 634.90 | 36967.63 ± 718.80 | 27195.19 ± 1124.44 | 90188.35 ± 1938.00 | 45274.26 ± 486.52 | 71432.46 ± 1099.31 | 58920.77 ± 606.59 | 150894.69 ± 4469.85 | 135640.02 ± 3177.38 | 162011.88 ± 2658.68 | 96592.69 ± 1348.65 |
| Propyl acetate | 780 | 725 | 784;730 | 15459.93 ± 515.18 | 4679.12 ± 65.49 | 8878.81 ± 205.19 | 27362.30 ± 1741.83 | 31652.85 ± 1215.02 | 1654.82 ± 44.67 | 16620.05 ± 467.32 | 43846.79 ± 353.33 | 8527.87 ± 369.72 | 831.51 ± 66.02 | 1309.27 ± 54.87 | 1365.19 ± 51.09 |
| Methyl pentanoate | 894 | 834 | 885;846 | 215.94 ± 9.93 | 0.00 | 55.14 ± 38.59 | 324.02 ± 18.56 | 569.86 ± 21.56 | 0.00 | 176.80 ± 12.54 | 724.41 ± 10.31 | 169.13 ± 13.19 | 0.00 | 0.00 | 0.00 |

Table S9. Entropy-weighted TOPSIS results for the 12 kiwi wines.

| **Number** | **D+** | **D-** | **C** | **Rank** |
| --- | --- | --- | --- | --- |
| HY-DPL | 0.032474205 | 0.070148077 | 0.683556002 | 1 |
| JY-QPL | 0.040073185 | 0.066898245 | 0.625384228 | 2 |
| JY-QPJ | 0.039963114 | 0.064307891 | 0.616738000 | 3 |
| XX-DPL | 0.049014355 | 0.063672606 | 0.565039697 | 4 |
| JY-DPJ | 0.048760394 | 0.057602548 | 0.541565949 | 5 |
| XX-QPL | 0.066552352 | 0.069730095 | 0.511658665 | 6 |
| HY-DPJ | 0.069101910 | 0.068442156 | 0.497601664 | 7 |
| JY-DPL | 0.061312957 | 0.060114012 | 0.495063100 | 8 |
| HY-QPJ | 0.062305858 | 0.056579832 | 0.475917933 | 9 |
| HY-QPL | 0.062162271 | 0.055058000 | 0.469696915 | 10 |
| XX-QPJ | 0.068634352 | 0.054349562 | 0.441924151 | 11 |
| XX-DPJ | 0.058212298 | 0.045510344 | 0.438769617 | 12 |

Note: D+ and D- denote Euclidean distances to the positive and negative ideal solutions, respectively. C denotes relative closeness. Treatments are ranked across all 12 combinations in descending order of C.

**Fig. S1** OPLS-DA score plot based on E-nose data

**Fig. S2** Permutation model validation plot

**Fig. S3** Variable importance in projection (VIP) scores obtained from the PLS model.

**Supplementary References**

Wang, G., Jing, S., Song, X., Zhu, L., Zheng, F., & Sun, B. (2022). Reconstitution of the flavor signature of Laobaigan-type Baijiu based on the natural concentrations of its odor-active compounds and nonvolatile organic acids. Journal of Agricultural and Food Chemistry, 70(3), 837–846. <https://doi.org/10.1021/acs.jafc.1c06791>

Gemert, L. J. (2011). Odour thresholds: Compilations of odour threshold values in air, water and other media (2nd ed.). Oliemans Punter & Partners BV.

Liu, X. S., Liu, J. B., Yang, Z. M., Song, H. L., Liu, Y., & Zou, T. T. (2014). Aroma-Active Compounds in Jinhua Ham Produced With Different Fermentation Periods. *Molecules*, 19(11), 19097-19113. <https://doi.org/10.3390/molecules191119097>
